# Translating single-neuron axonal reconstructions into meso-scale connectivity statistics in the mouse somatosensory thalamus

**DOI:** 10.3389/fninf.2023.1272243

**Published:** 2023-12-01

**Authors:** Nestor Timonidis, Rembrandt Bakker, Mario Rubio-Teves, Carmen Alonso-Martínez, Maria Garcia-Amado, Francisco Clascá, Paul H. E. Tiesinga

**Affiliations:** ^1^Neuroinformatics Department, Donders Centre for Neuroscience, Radboud University Nijmegen, Nijmegen, Netherlands; ^2^Institute of Neuroscience and Medicine (INM-6) and Institute for Advanced Simulation (IAS-6) and JARA BRAIN Institute I, Jülich Research Centre, Jülich, Germany; ^3^Department of Anatomy and Neuroscience, School of Medicine, Autónoma de Madrid University, Madrid, Spain

**Keywords:** single-cell morphology, VPM, somatosensory cortex, topography, connectomics, Coherent Point Drift, t-SNE, projection motifs

## Abstract

Characterizing the connectomic and morphological diversity of thalamic neurons is key for better understanding how the thalamus relays sensory inputs to the cortex. The recent public release of complete single-neuron morphological reconstructions enables the analysis of previously inaccessible connectivity patterns from individual neurons. Here we focus on the Ventral Posteromedial (VPM) nucleus and characterize the full diversity of 257 VPM neurons, obtained by combining data from the MouseLight and Braintell projects. Neurons were clustered according to their most dominantly targeted cortical area and further subdivided by their jointly targeted areas. We obtained a 2D embedding of morphological diversity using the dissimilarity between all pairs of axonal trees. The curved shape of the embedding allowed us to characterize neurons by a 1-dimensional coordinate. The coordinate values were aligned both with the progression of soma position along the dorsal-ventral and lateral-medial axes and with that of axonal terminals along the posterior-anterior and medial-lateral axes, as well as with an increase in the number of branching points, distance from soma and branching width. Taken together, we have developed a novel workflow for linking three challenging aspects of connectomics, namely the topography, higher order connectivity patterns and morphological diversity, with VPM as a test-case. The workflow is linked to a unified access portal that contains the morphologies and integrated with 2D cortical flatmap and subcortical visualization tools. The workflow and resulting processed data have been made available in Python, and can thus be used for modeling and experimentally validating new hypotheses on thalamocortical connectivity.

## 1 Introduction

Direct, orderly communication between cell populations in distant parts of the brain is made possible by long-range projection neurons (LRPN). The LRPN axons create the brain-wide circuits that enables the highly integrated brain functioning that makes perception, memory, consciousness and skilled movement possible (Sherman, 2016). Depending on their specific branching patterns, LRPN axons can establish parallel (point-to-point) or convergent/divergent (point-to-multi-point) connections, in unidirectional or reciprocal patterns. Importantly, the functional impact of the signals carried by LRPN axons on other cell populations depends on the precise target distribution and the number of their synapses, which is highly diverse and specific for each LRPN type (Clascá, 2022).

Thalamic projection neurons are a key and well-characterized group of LRPN situated deep within the brain. Some of them act as the gateway for sensory information to the cerebral cortex. These neurons cluster together forming the so-called first order relay nuclei of the thalamus, and their axons innervate the ipsilateral cerebral cortex in a focal orderly fashion. One of these nuclei is the ventral posteromedial nucleus of the thalamus (VPM). The VPM projection neurons receive monosynaptic inputs from the trigeminal complex of the brainstem carrying mechano- and nociceptive signals originating in the face and mouth, and relay them to the somatosensory areas of the cortex. In many rodents, this system includes a specialized subsystem that enables these animals to use their motile mystacial vibrissae as high-resolution haptic tactile probes (Bosman et al., 2011).

A number of studies have revealed interesting properties of VPM LRPN neurons and of other thalamic nuclei (Waite, 1973; Saporta and Kruger, 1977; Ito, 1988; Sugitani et al., 1990). The first observation is that these neurons are topographically organized with respect to both their soma location and their axonal terminals in the barrel cortex. The second observation is the existence of region-specific heterogeneous projections from a single nucleus (Han et al., 2018; Muñoz-Castañeda et al., 2021), which suggests the organization of thalamic projections into higher order connectivity motifs (Clascá et al., 2012). These findings indicate that morphological diversity can be found even in a first-order nucleus, which has traditionally been considered to be very homogeneous in terms of the morphology of its neurons.

The concept of motifs plays a prominent role in neuroscience. To provide proper context for the more mesoscopic motifs introduced here, we discuss these prior uses of motifs. In a random network, the directional connections from one neuron to another neuron are made with a certain probability *p*. The probability for an arbitrary pair to have a single connection is *p*. The probability for a reciprocal connection is *p*^2^. However, when in a network this probability is significantly higher than *p*^2^ than we say there are connectivity motifs. This usage (Song et al., 2005) is derived from earlier work in genetic networks (Milo et al., 2002) and has since been applied in many other works (Perin et al., 2011; Vasquez et al., 2013). Another related concept is circuit motifs, which applies to networks comprised of multiple neuron types, such as pyramidal cells, and multiple inhibitory neurons, such as parvalbumin (PV), somatostatin (SOM) and vasoactive intestinal peptide (VIP) expressing neurons and where there is a pattern of connectivity, such as that SOM projects to VIP and PV, but PV does not project to SOM (Pfeffer et al., 2013). Circuit motifs can be related to computations they perform (Womelsdorf et al., 2014) and oscillation frequencies they index (Ter Wal and Tiesinga, 2021). This is in contrast with the projection pattern motifs characterized here, which are related to the presence of higher order structure in projection patterns from individual neurons within a source brain area to multiple target brain areas (Han et al., 2018). This higher order structure should also be statistically significant, meaning that it deviates from the null hypothesis of exclusive first-order projections from a source to target brain areas. According to the null hypothesis, the probabilities of source neurons targeting two or more distinct target brain areas are statistically independent of each other. Significant deviations from this assumption can be considered as evidence for the higher-order organization of LRPN neurons into motifs of projection patterns that are not random.

However, these qualities have been difficult to analyze in experimental data, despite a tremendous development of connectomics approaches in the past decades (Cazemier et al., 2016). On the one hand, it remains impossible to extract single-cell morphologies from a neural population using classical tracing techniques (Oh et al., 2014; Harris et al., 2019) or diffusion tensor imaging (Calabrese et al., 2015). On the other hand, electron microscopy (Kasthuri et al., 2015) and direct synaptic labeling (Druckmann et al., 2014) do not cover a large enough volume to appropriately quantify the morphology of LRPN neurons. These studies instead focus on local circuits, that is, local intracortical projections.

Fortunately, recent developments in automated tissue-to-volume reconstructions of single neurons based on fluorescence micro-optical sectioning tomography (fMOST) (Li et al., 2010) or light-sheet fluorescence microscopy (Economo et al., 2016), enable to image these morphologies at light-microscopic resolution. Despite this progress, characterizing projection motifs in hundreds of single neurons is still challenging and labor intensive. As a result, the sample sizes, even for the most intensively studied LRPN groups are still small, since thousands of neuronal reconstructions are needed to reach a nucleus-wide coverage similar to the mesoscale population experiments by Oh et al. (2014) and Harris et al. (2019). An additional challenge is that topographic maps in relation to high-order connectivity patterns of the brain have not yet been quantitatively characterized, which makes it hard to generalize observations from related data and refine them into mathematically rigorous approaches (Battiston et al., 2020; Bick et al., 2022).

While the existing large-scale repositories have made available large amounts of single-neuron axonal reconstructions (Han et al., 2018; Winnubst et al., 2019; Muñoz-Castañeda et al., 2021; Peng et al., 2021; Gao et al., 2022), they nevertheless have failed to live-up to expectation because each of them were used in isolation. Therefore, data integration from multiple repositories into a collaborative infrastructure, registered into a common reference space and combined with cutting edge neuroinformatics tools for statistical analysis, is necessary for providing context and insight in the morphological distribution and structural organization of thalamic nuclei.

Moreover, to characterize the projection diversity of a neuronal population, we need to go beyond comparing different neurons solely on the basis of their soma position and projection targets. If two neurons with a different soma position in the same source nucleus differ in the targeted areas, there is a number of possible assumptions that can account for that difference. One assumption is that the source region is heterogeneous, with these two neurons belonging to different cell-types, which have different morphologies. The second assumption is that they belong to the same cell-type and that the differences in their projections are due to the position they occupy along the topographical axis within that source region. The last assumption is that the difference is a by-product of the anatomical parcellation scheme, such as the Allen Reference Atlas (ARA) or the Paxinos & Franklin (PF) atlas (Paxinos and Franklin, 2019), which implies that in a different parcellation scheme the two neurons would be classified as targeting the same area. For the first and second assumptions, topographical and morphological analyses will have to be carried out, respectively. For the third assumption, neurons would have to be compared under different parcellation schemes, with the correct one decided by the expert.

For a morphological analysis, morphometrical measures (morphometrics) are used to describe different axonal or dendritic morphological properties of neurons and thus serve as useful features for clustering neurons into types with distinct morphological characteristics (Laturnus et al., 2020; Walker et al., 2022). However, classical morphometrics are inadequate to capture the local topology and geometry of axonal branches and terminals. The reason is that morphometric analyses rely on global pre-defined measurements and do not capture pair-wise local variations between neurons, which obscures the estimation of neuronal variability and biases the global morphometrics employed for this purpose (Kanari et al., 2018; Batabyal et al., 2020). Therefore, when estimating morphological diversity, one needs to take into account the entire axonal tree instead of only regions of interest or pre-computed morphometrics.

To address the aforementioned issues, we have developed a workflow for translating ~300 VPM axonal morphologies into projection statistics with a twofold aim. We analyze the topographical correlations between the VPM and various somatosensory areas and identify distinct subpopulations inside VPM that differ in their connection motifs across the somatosensory areas. The projection statistics are measured according to reconstructed axonal morphologies of long-range projection neurons obtained from two publicly available high-throughput databases, namely *MouseLight* (Winnubst et al., 2019) and *Braintell* (Peng et al., 2021). We integrate data from both datasets to deal with the limited sample size of the individual datasets of reconstructed neurons, since together they comprise the most extensive publicly available collection of reconstructed neurons from the thalamus to date.

Moreover, we further characterize the morphological diversity of VPM neurons beyond their projection patterns and in relation to their topographical organization using a robust point-cloud alignment and registration approach called Coherent Point Drift (CPD) (Myronenko and Song, 2010). CPD can compute in an unbiased fashion the morphological distance of two different neurons based on their axonal morphology and anatomical coordinates that goes beyond a given anatomical parcellation. We use CPD to estimate the morphological distance between all possible neuronal pairs, which in turn provides us a distance criterion for defining gradients or clusters that characterize the morphological diversity of VPM. To assess the robustness of CPD-based morphological clusters or types, we overlay them with the corresponding projection types and align them with their topographical orientation along the dorsal-ventral axis. This allows us to analyze the morphologies simultaneously in relation to their topographical, morphological and projection properties. Finally, we implement a number of 2D cortical flatmap and VPM spatial plots for visually inspecting the newly characterized and diversified morphologies in the spatial context of the cortical surface and the VPM nucleus. This analysis could be in principle extended to other types of thalamic projection cells which are structurally more diverse, to help a comprehensive and objective delineation of projection neuron types.

## 2 Materials and methods

### 2.1 Data retrieval and neurons reunited portal

Over the past 5 years, five repositories of fully traced neurons have been released: the “*MouseLight*” database (1,500 neurons, Winnubst et al., 2019), the “*Braintell*” database (1,700 neurons, Peng et al., 2021), the “prefrontal” database (6,300 neurons, Gao et al., 2022) and the smaller collections of visual cortex neurons (46 neurons, Han et al., 2018) and primary motor cortex neurons (38 neurons, Muñoz-Castañeda et al., 2021). These five datasets together comprise currently the most extensive collection of single-neuron long-range projection data, bridging the gap between local microcircuits and brain-wide axonal projections.

**Figure 1:**
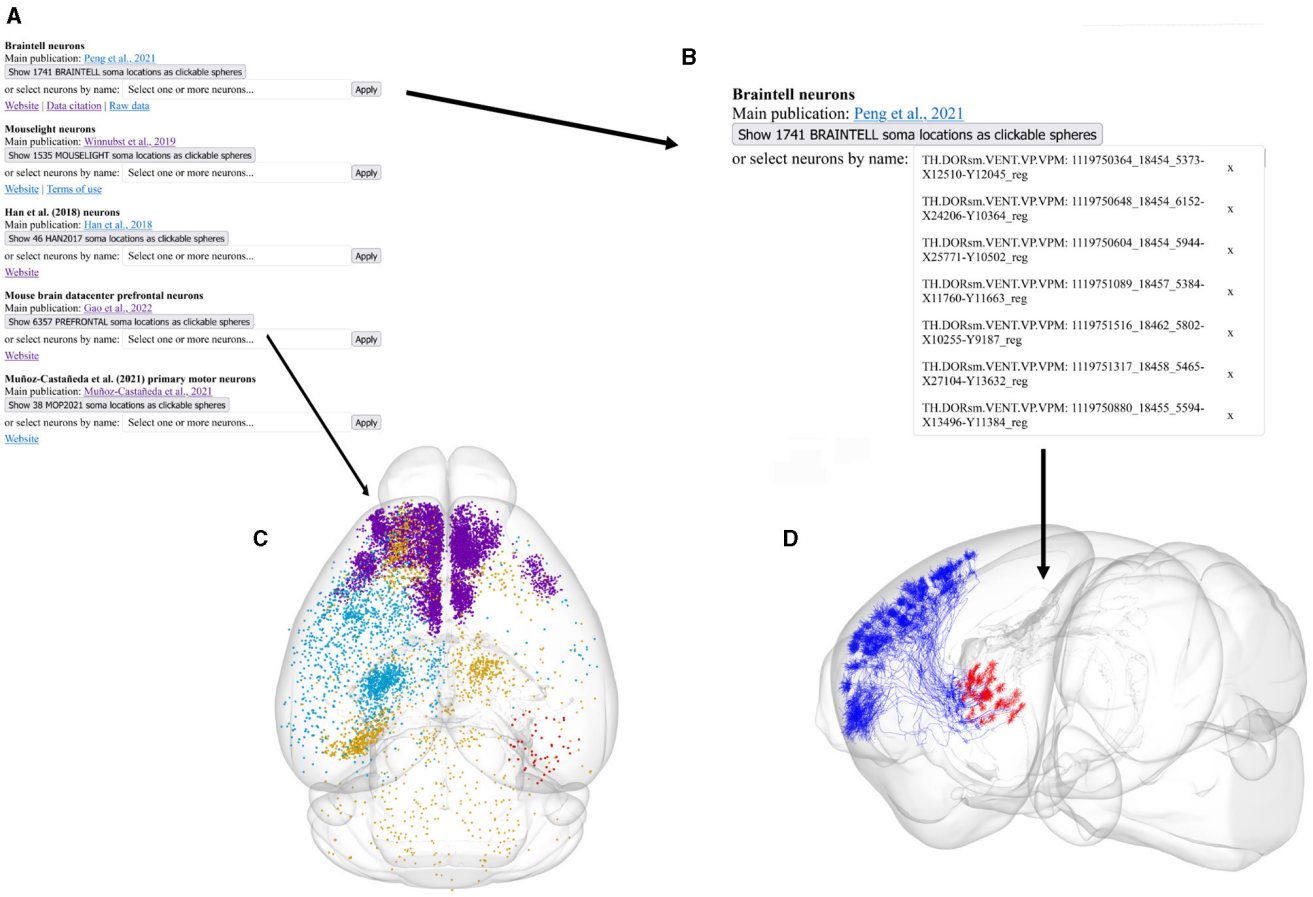
Illustration of the different parts of the *Neurons Reunited Portal*. **(A)** Partial view of the portal webpage indicating all available databases for visualization and analysis. For each database, the following format has been adopted: the first line describes the name of the database, the second line provides a hyperlink to the respective online publication, the third line is a button enabling the visualization of the somata of all related neurons, the fourth line is a search bar that allows users to search for related neurons with a simple click, and the last line provides hyperlinks for related websites, terms of use, raw data and data citation, if available. **(B)** Inset of the webpage zooming in on the search bars illustrating how a user can search neurons by database and source brain area. In this example, the keyword “VPM” has been entered in the *Braintell* search bar. As a result, a list of VPM neurons has appeared to help the user with selecting the appropriate subset of neurons. The acronyms used to characterize each neuron are a combination of the acronyms of taxonomical subdivisions to which the source area of the neuron belongs according to ARA, together with the naming convention provided by the home database of the neuron. For instance, the top acronym appearing on the search bar refers to thalamus (TH), sensory-motor cortex related (DORsm), ventral group of the dorsal thalamus (VENT), ventral posterior complex (VP) and ventral posteromedial nucleus (VPM), while the remaining acronym is the name id of the neuron given by *Braintell*. **(C)** Visualization of all available morphologies that are hosted in the portal as clickable spheres. The neurons are color-coded based on their respective database: the light blue color corresponds to *Braintell*, orange corresponds to *MouseLight*, red corresponds to the visual cortex database, purple corresponds to the prefrontal database and light green corresponds to the motor cortex database. By clicking on a cell body, its respective morphology gets rendered in a similar fashion to panel **(C)**. The brain template is in horizontal view. **(D)** Visualization of the selected VPM morphologies using the SBA Composer visualization tool. The red and blue colors correspond to dendritic and axonal, segments, respectively. The brain template is in a sagittal view that is slightly rotated around the superior-inferior axis such that the relationship between axons, dendrites and soma is more clearly visible.

We thus decided to take the opportunity and facilitate access to the community with the *Neurons Reunited Portal*: a unified access portal to these resources (see Table 1). The website can be used to browse all neurons, and it has a REST application programming interface (API) to pre-select a set of neurons for visualization (see Figures 1A, B). *Neurons Reunited Portal* is similar to Neuromorpho (Ascoli et al., 2007) in terms of storing neuronal morphologies, with the main difference that all morphologies are by default registered to the common coordinate framework (CCF) v3.0, which allows their direct use in data integration frameworks and spatial statistical analyses such as the ones presented in this work.

**Table 1:** Hyperlinks for websites, tool descriptions and format descriptions related to our analysis.

**NeuronsReunited morphology database viewer**	**https://neuroinformatics.nl/HBP/neuronsreunited-viewer/**
NeuronsReunited cortical flatmap viewer	https://neuroinformatics.nl/HBP/allen-flatmap/
Repository of our Code on the EBRAINS Collaboratory	https://wiki.ebrains.eu/bin/view/Identity/#/units/all:projects:hbp_pp:neuronsreunited
Github Code Repository for the analysis performed in this work	https://github.com/ntimonid/morphological_embedding
Github Code Repository for mesoscale statistics	https://github.com/ntimonid/Mesoscale_Extractor
Github Code Repository for finding similar neurons	https://github.com/ntimonid/Neuron_Aligner
Allen Institute for Brain Science	https://alleninstitute.org/
AMBCA repository (Oh et al., 2014; Harris et al., 2019)	https://help.brain-map.org/display/mouseconnectivity/Documentation
CCF v3.0 (Wang et al., 2020)	http://connectivity.brain-map.org/3d-viewer
MouseLight database (Winnubst et al., 2019)	https://ml-neuronbrowser.janelia.org/
Braintell database (Peng et al., 2021)	https://braintell.org/seu-allen/index.html
Cortical flatmap templates (Knox et al., 2018)	https://download.alleninstitute.org/informatics-archive/current-release/mouse_ccf/
NumPy	https://numpy.org/
Matplotlib	https://matplotlib.org/
NIfTI files	https://nifti.nimh.nih.gov/
JSON files	https://en.wikipedia.org/wiki/JSON
SBA Composer (Bakker et al., 2015)	https://sba-dev.incf.org/composer/index.php
MorphoPy library (Laturnus et al., 2020)	https://morphopy.readthedocs.io/en/latest/
Coherent Point Drift library (Myronenko and Song, 2010)	https://github.com/siavashk/pycpd
Xml dom minidom library	https://docs.python.org/3/library/xml.dom.minidom.html
Extensible 3D script library	https://www.x3dom.org/

See main text for details.

Moreover, the registration of morphologies in CCF enables their visualization in the Scalable Brain Atlas (SBA) composer 3D visualization tool (Bakker et al., 2015), which works as a back-end for the portal. SBA composer can simultaneously visualize all morphologies that are selected by a user, overlaid on a 3D mesh of the CCF mouse brain template (Figures 1C, D). In addition, by clicking on the various brain regions that each neuron originates from or traverses, SBA can render the respective region as a semi-transparent mesh, as well as color-code the laminar distribution of the neurons with different colors, which can significantly enhance the identification and characterization of different sub-types of neurons. Beyond the selection and visualization of morphologies, we have connected the portal with offline Python-based script modules to search for neurons with particular properties, such as “originating in the thalamus and mainly targeting the primary visual cortex” or with similar morphologies as a number of pre-selected neurons. These enhanced search facilities are part of the workflow presented in this work and have been integrated through bi-directional API interfaces that establish a communication between a Jupyter Notebook analyzing the selected neurons and the *Neurons Reunited Portal*.

From the above datasets, we stored the complete morphological reconstructions from *MouseLight, Braintell* and the visual and motor cortex databases. For the prefrontal cortex database, we currently store only the neuronal soma positions. The complete prefrontal reconstructions will be available in the next upgrade of the portal. The original file format of all obtained morphologies is swc (Stockley et al., 1993), which is the de facto format for morphological reconstructions. To store the morphologies in the portal we converted their respective swc files to *JavaScript Object Notation* (JSON) file formats. The reason for file conversion is because JSON provides a greater degree of flexibility in using Python-related libraries for the analysis described in this work (see Supplementary Section 1.1 for more details regarding the data analyzed in this work).

**Figure 2:**
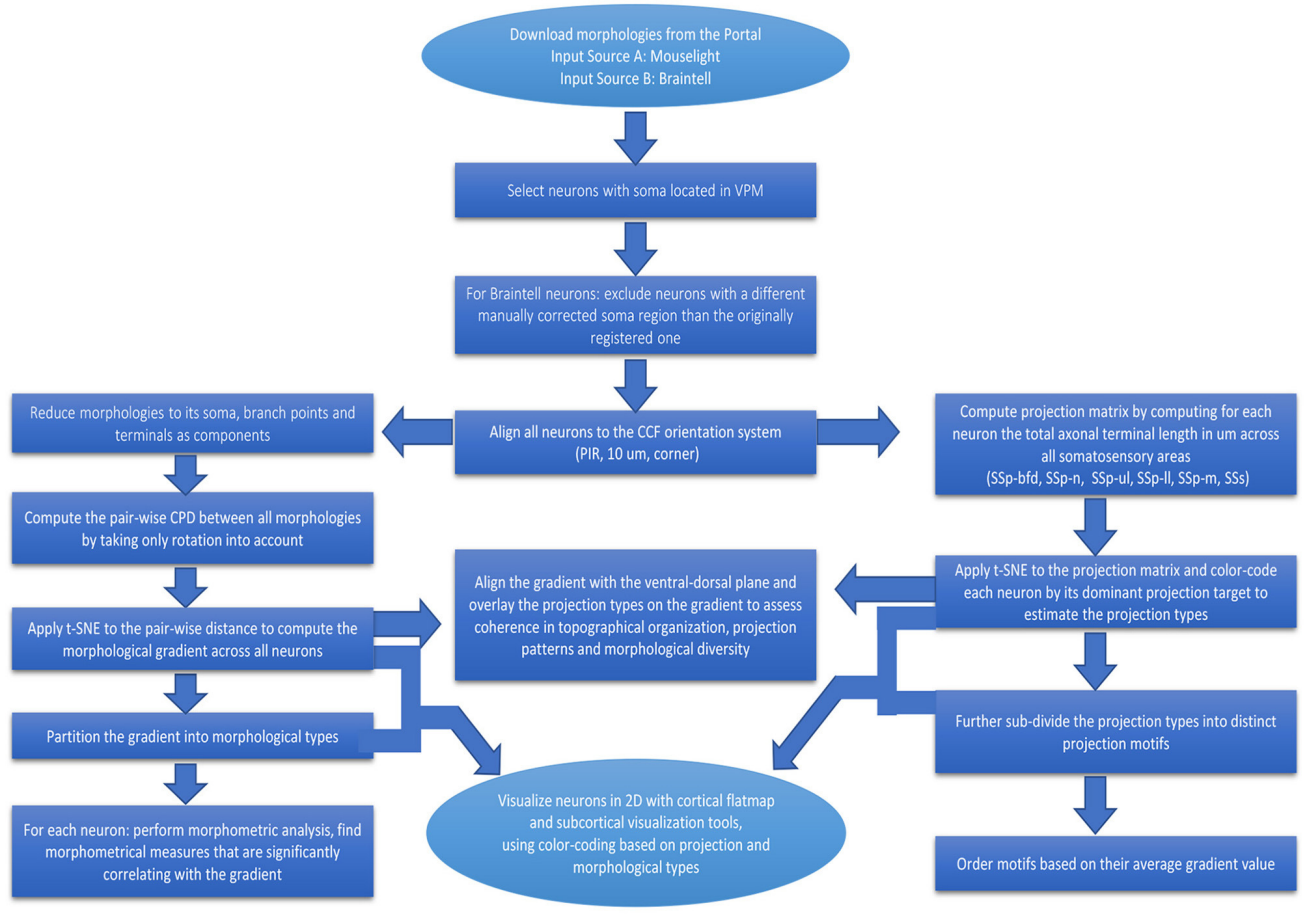
Flowchart providing a step-by-step description of our workflow for the analysis of LRPN neurons. Boxes correspond to the various steps comprising the workflow, from pre-processing to analysis and visualization of the data, and arrows represent the directionality of performed actions.

In the workflow described in this study, we analyzed data from two sources of reconstructed neurons from the large online repositories of *MouseLight* and *Braintell*. We did not include data from the other databases since they do not contain VPM neurons, which are the focus of this study. A major reason for considering VPM instead of other thalamic nuclei is that it is comprised of a well-studied group of LRPN neurons. Another reason is that it is the most densely sampled nucleus in the thalamus and the second most densely sampled area across the entire mouse brain, when considering all neuronal reconstructions from the *MouseLight* and *Braintell* databases together: the first area is the secondary motor cortex (MOs) with 352 neurons and the third area is the caudoputamen (CP) with 256 neurons. In the following sections, we describe different parts of the analysis we conducted to better comprehend the VPM neuronal diversity (see Figure 2 for a schematic description of the various steps comprising the workflow).

### 2.2 Data pre-processing

We retrieved data by using an API to download reconstructed neuronal morphologies from the *Neurons Reunited Portal*, from which 27 were from *MouseLight* and 256 were from *Braintell*. The morphologies were downloaded in the JSON file format, for reasons described in Section 2.1. Neurons with a soma position not located in the VPM were filtered out prior to the analysis.

When selecting neurons from the *Braintell* database, we first had to use a strict selection criterion regarding the registration procedure. For each *Braintell* neuron, manual corrections were made by Peng et al. (2021) to provide its correct soma area, in order to mitigate errors caused by their fully automated reconstruction pipeline (see Supplementary Section 1.1). However, the corrected 3D coordinates of the soma of each neuron were not released after correction, which would have allowed users to fully utilize the neuron in analyses such the one described here. Therefore, we excluded 26 *Braintell* morphologies with a manually corrected soma region that did not match the original one. We considered these cases to be potentially misregistered or not originating in the VPM. We thus retrieved 257 morphologies in total for analysis, after applying this selection criterion for all VPM *Braintell* neurons and after obtaining all VPM *MouseLight* neurons.

Each morphology was represented as a list of two arrays in which the first corresponds to the anatomical coordinate of each point in CCF and the second contains relevant information about each point, such as its identity (soma, dendrite, axon), its index in the first list and the index of its parent in the list. The parent of a point corresponds to its direct ancestor in the morphological tree, which could be either an axonal branch, dendritic branch or the soma. We label the first array as the “point” array, since its elements are points in 3D space, and the second array as the “line” array, since its elements are lines that connect the points from the first array.

Prior to analysis, we had to ensure for all neurons that the orientation of the coordinate system was the same as for CCF (anterior-posterior, superior-inferior, left-right with origin at the anterior-superior-left corner), by means of an affine transformation. We represented all morphology coordinates at a 10 μm resolution, which is the highest available resolution for the ARA template. First, *Braintell* neurons are already oriented at PIR (anterior-posterior, superior-inferior, left-right) at 1 μm resolution, with a small fraction having 25 μm resolution. We thus re-scaled the anatomical coordinates of these two *Braintell* groups by a factor of 10 and 0.4, respectively. Second, *MouseLight* neurons are oriented at LIP (right-left, superior-inferior, anterior-posterior, corner origin) at 1 μm resolution. For the anatomical coordinates of each neuron, we switched the first and the third coordinate, reflected the third coordinate and re-scaled all coordinates by a factor of 10.

Following the transformation of all neurons to the same reference space, we could now represent each reference neuron as a 3D point cloud. In addition, the parent-child relationship between points allowed us to treat the point-cloud as a morphological tree and hence perform a number of morphometric analyses to it, such as estimating for each axonal segment its path length, radial distance from the soma, branching order, angles, width and height, as well as the number of terminals, thickness and total volume or surface covered.

To alleviate the cost of morphological analysis, we reduced each morphology to its topological minor, which means that it was only represented by its soma, axonal branches and terminals. To identify the axonal terminals, we found axonal points that had no children points. We then extracted the branch of a given terminal by backtracking from the terminal point its ancestor points until a branching point was reached. Therefore, we comprised a list of the axonal terminal branches of each neuron and we integrated them with their branching nodes to preserve the morphological tree structure. The topological minor form allowed us to significantly reduce the computation time of morphological analyses, since it had an on average 30-fold (15,872–411) decrease in number of points used to represent each morphology.

While the above pre-processing steps treated the neurons as morphological trees, we had to create a representation that allowed us to quantify the projection patterns of neurons at the meso-scale level of anatomically distinct subdivisions of the somatosensory cortices, in order to distinguish types of neurons with distinct projection patterns. We therefore defined for each neuron its somatosensory projection targets by estimating the total axonal terminal branch length in μm for each sub-region in the somatosensory cortex: primary somatosensory cortex whiskers/barrel-cortex (SSp-bfd), nose (SSp-n), mouth (SSp-m), upper limb (SSp-ul), lower limb (SSp-ll) and supplemental somatosensory area (SSs). That allowed us to translate morphologies into a connectivity matrix that could be used to define neuronal projection types (see Supplementary Section 1.4 for more details).

### 2.3 Searching for similar neurons using the Coherent Point Drift

**Figure 3:**
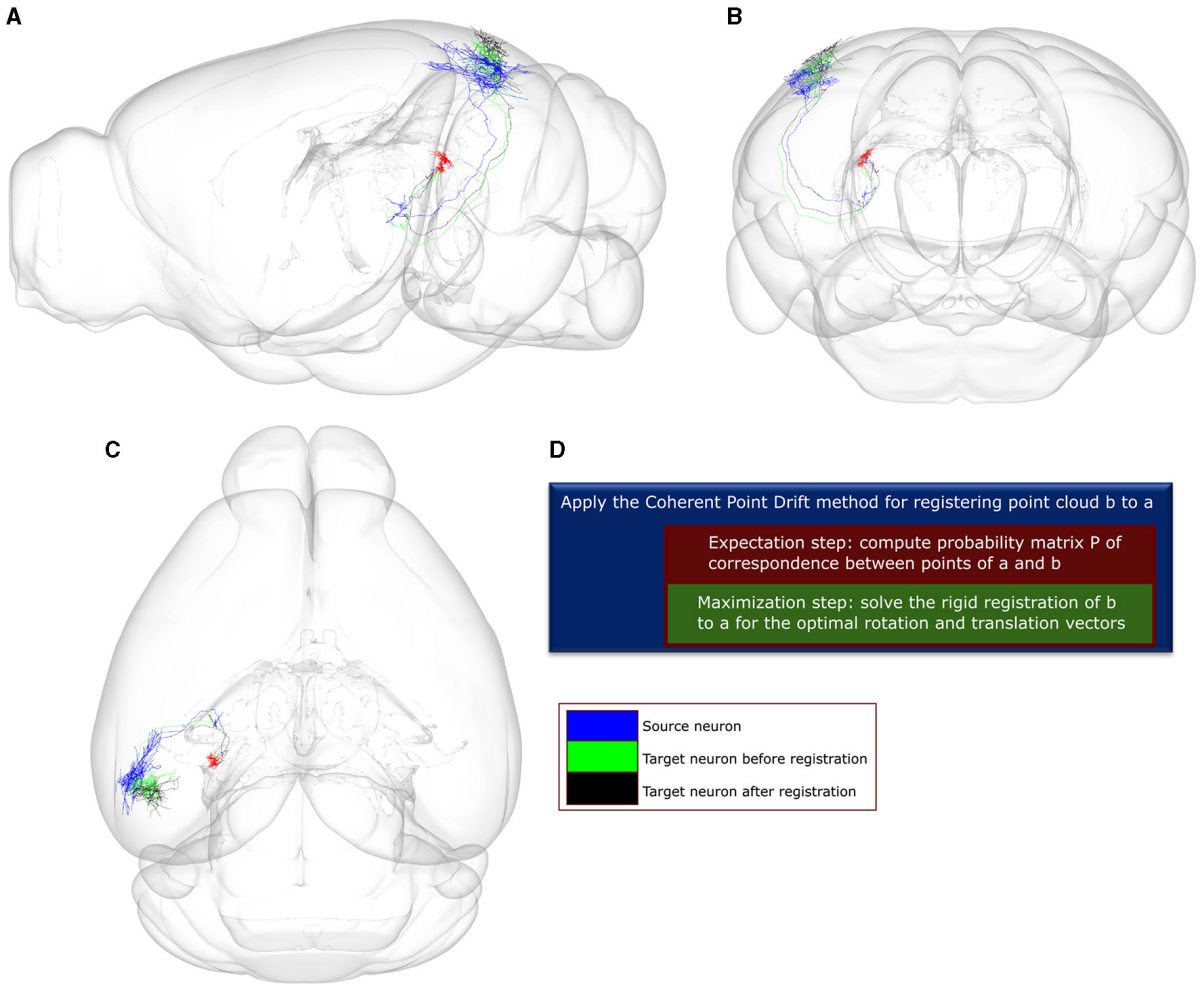
A visualization of the point-to-point correspondence and rigid registration procedure using the *Coherent Point Drift* (CPD) algorithm, with a use-case involving two example neuronal morphologies a and b that originate in the posterior nucleus of the thalamus (PO). In this use-case, the CPD algorithm is used to find the corresponding points between a and b and subsequently register b to a using rotation and translation. The error for registering b to a is quantified by the mean square error (MSE), which is estimated between each point in b and its corresponding point in a. After 0 and 60 iterations, the MSE is 4.4e+04 and 1.66e+04, respectively. **(A–C)** Sagittal, coronal and horizontal views of an anatomical mouse brain template based on CCF, to which both neurons have been registered and are spatially overlaid. Green and black colors: axonal branches of neuron b after 0 and 60 rounds of iteration. Blue color: axonal branches of neuron a. Red color: soma locations of both neurons. **(D)** Description of the CPD algorithm. The high throughput axonal reconstructions have been retrieved from the *Braintell* repository (Peng et al., 2021). The visualizations were made using the SBA Composer 3D visualization tool (Bakker et al., 2015).

We then applied the Coherent Point Drift (CPD) method (Myronenko and Song, 2010) to compare all neuronal pairs through a two-step registration process. Given a source and target neuron, CPD finds the missing correspondences between points of the two neurons by minimizing the negative log-likelihood that the point cloud of the source neuron was sampled from the distribution of the target neuron, as modeled by a Gaussian Mixture Model. Subsequently, CPD applies a rigid transformation of the target point-cloud to the source one (Figure 3). The algorithm converges when one of two criteria is met: the algorithm reaches an upper bound of 60 iterations, or the difference between consecutive log-likelihood values falls below the tolerance of 0.001. The values for both criteria were selected to be as strict as possible while achieving convergence under 20 s given an average size of 400 points comprising of the point-clouds used as pairs for the CPD registration. Following convergence, we selected as a neuronal match to each source neuron the target neuron that minimized across all pairs the mean square error (MSE) of registration to the source.

### 2.4 General methodology for identifying sub-types of neurons

We have produced a statistical description of the projection motifs of a sensory first-order thalamic nucleus, namely the ventral posteromedial nucleus (VPM) of the thalamus. We characterized neurons in terms of their dominant projection target (Section 2.4.1), and subdivided these groups based on their pattern of projections to secondary areas (Section 2.4.2) as well as on the similarity of the morphology (Section 2.4.3).

#### 2.4.1 Distinguishing projection types

**Figure 4:**
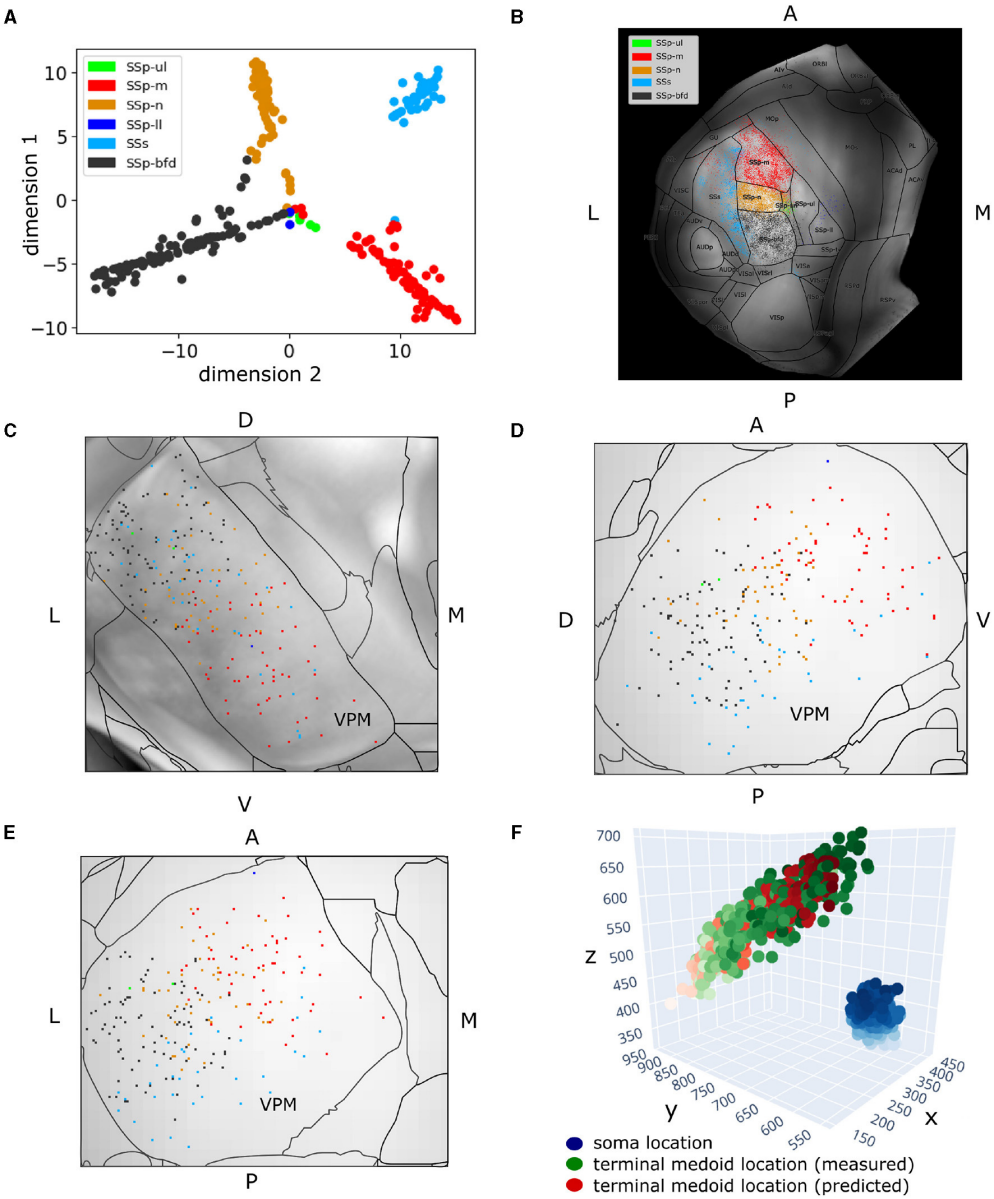
Two hundred and fifty-seven VPM neurons exhibit topographical organization in both their soma and axonal terminal location. **(A)** Scatter plot illustrating a 2D embedding of the VPM neurons based on their projection patterns across six somatosensory areas (Section 2.4). The projection of each neuron to a given area is defined as the total length of axonal branches that terminate in that area. Each acronym denotes a somatosensory area and the respective color is used to label neurons that have that area as their dominant projection target. The colors are consistent throughout the figure. The *x*- and *y*-axes correspond to the projection values of each neuron in the first and second dimension of the embedded space. **(B)** Dorsal cortical flatmap overlaid with anatomical borders illustrating the axonal termination patterns of the VPM neurons (13.6 × 13.6 mm along the anterior posterior and left-right axes). **(C–E)** Visualization of neuronal cell bodies in VPM across coronal **(C)**, sagittal **(D)** and horizontal **(E)** planes (1.37 × 1.54 × 1.62 mm along the anterior-posterior, superior-inferior and left-right axes, respectively). The letters at the side of each panel denote the plot orientation. **(F)** 3D scatter plot demonstrating the topographical correlation between the soma position of VPM neurons and their corresponding axonal termination location. Blue color: VPM soma position, with the light-to-dark contrast reflecting the soma position along the ventral-dorsal and medial-lateral axes. Green color: measured axonal terminals, with the light-to-dark contrast reflecting the terminal position along the anterior-posterior and lateral-medial axis. Red color: predicted axonal terminals calculated based on VPM soma position using a least-squares model (see Section 3), with light-to-dark contrast being the same as for the green points. The MSE for reconstructing the measured terminals with this model was 931. The orientation of each axis is shown on its side and follows the RAS orientation: *x*-axis corresponds to the left-right, *y*-axis corresponds to the posterior-anterior axis, and *z*-axis corresponds to the inferior-superior axis of CCF.

Prior to delineating neurons based on their projection or morphological properties, we applied the t-SNE dimensionality reduction technique (van der Maaten and Hinton, 2008) to create a 2D embedding of the VPM neurons based on their projection patterns across the six somatosensory areas. We thus visualized the data in two dimensions and identified the main sources in axonal variation across the cortex (Figure 4A). Each neuron in its embedded representation was assigned a color that corresponded to the brain area that was its dominant projection target (Figures 4B–E). The dominant projection target of a neuron was defined as the somatosensory area that receives the majority of axonal terminal branches of the neuron compared to the other somatosensory areas. This resulted in a fast, greedy and parameter-free clustering approach which can be applied to quickly pinpoint the dominant projection patterns of the available thalamocortical (TC) morphologies.

To quantify the topographical correlation between the soma positions of VPM neurons and their axonal terminals in the somatosensory cortices, we performed a least-squares regression to fit the soma position of these neurons to the medoids of their axonal terminals. We selected the medoids instead of the centroids to represent the multiple targets that a soma can have, because the medoid measure provides an actual target location that is in the data rather than a location between two or more separated termination domains. By treating the regression coefficient between the two coordinate systems as a rigid transformation matrix, we extracted the corresponding rotation by decomposing the transformation matrix using polar decomposition. We then computed the Euler angles in the *x, y*, and *z* axes from the rotation matrix to numerically assess the 3D rotation describing the topographical relationship between the VPM soma positions and their cortical targets (Figure 4F).

**Figure 5:**
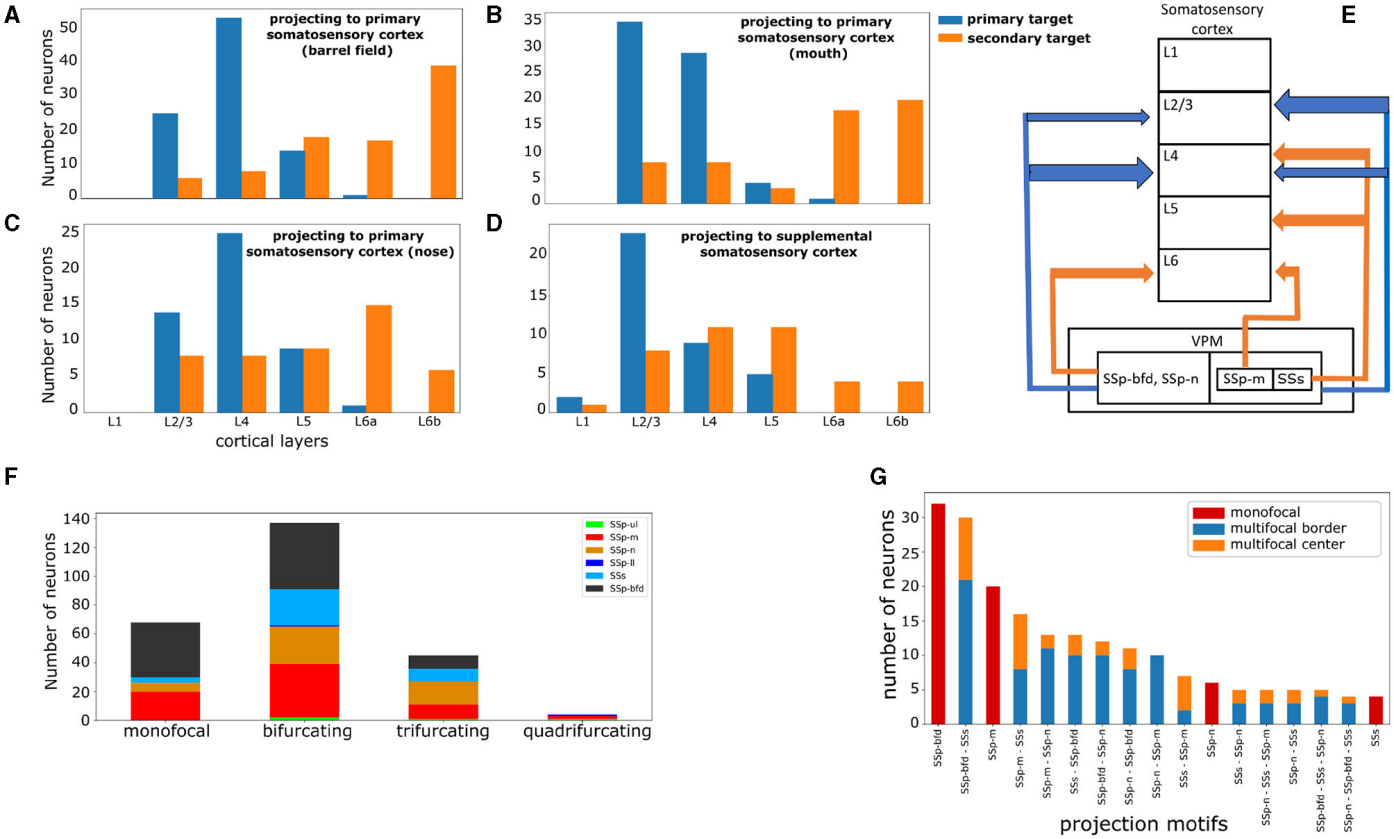
VPM neurons exhibit distinct laminar projection patterns and can be further subdivided by higher order projection motifs. **(A–D)** Four plots highlighting the cortical layer distribution of the first and second most dominantly targeted area for each VPM neuron, with the first area being either the barrel field **(A)**, mouth **(B)**, nose **(C)**, or supplemental **(D)** somatosensory area. *x*-axis: cortical layers. *y*-axis: number of neurons projecting to a given layer with the highest fraction of terminal length in their first (blue color) and second (orange color) most dominantly targeted area. **(E)** Schematic illustration that summarizes panels **(A–D)**. The vertical boxes represent cortical layers 1–6 and the nested horizontal boxes represent VPM with its subdivisions based on the projection types that we have delineated in this work. The arrows represent projections to a cortical layer and the arrow thickness represents the relative number of neurons projecting to that layer. The color-coding is the same as in panels **(A–D)** and it distinguishes primary from secondary projections. The sub-nesting of projection types indicates common projection patterns; SSp-bfd and SSp-n projecting neurons have common laminar projection patterns in both their primary and secondary targets, while SSp-m and SSs projecting neurons share only common primary laminar projection targets. **(F)** Distribution of neurons based on the total number of jointly targeted brain areas. The motifs exhibit first and higher order connectivity reaching up to three jointly targeted somatosensory areas. *x*-axis: categories of the different orders of connectivity: monofocal, bifurcating, trifurcating and quadrifurcating correspond to the targeting of one, two, three and four areas, respectively. *y*-axis: number of neurons belonging to each category. The color-coding is used to distinguish the different projection types and is consistent with Figure 4A, in which the projection types are first introduced. **(G)** Distribution of the unique projection motifs identified in this work that have been sorted in descending order by the number of neurons participating in each motif and color-coded by the dominant projection target area, with the color-coding as in Figure 4. The areas characterizing each motif are ordered in descending order by the number of axonal terminals received from a single-neuron. The red, blue and orange colors are used to distinguish neurons based on the topography of their projections. Red: monofocal neurons. Blue: multifocal neurons which project in the border between the dominant and the secondary target areas. Orange: multifocal neurons having at least four axonal terminals in the secondary target areas with at least 200 μm distance from the border between these areas and the dominant target area.

Lastly, we assessed the distribution of axonal terminal branch length per layer expressed in μm for each of the four somatosensory areas receiving the highest projection length (barrel field, mouth, nose and supplemental somatosensory area, see Figures 5A–E and Supplementary Figure 1), in order to understand the lamination patterns of each projection type.

#### 2.4.2 Distinguishing projection motifs

The subsequent analysis involved finding projection motifs. A motif was defined by the unique combination of brain areas targeted by the axonal terminals of a given neuron. We identified sub-types based on distinct projection motifs: monofocal neurons projecting to only one area, bifurcating ones projecting to two areas and smaller fractions projecting to more than two areas. For each of the above TC neurons, we obtained the anatomical distribution of their axonal terminals. To filter out weak connections, we estimated which cortical areas received at least five terminal branches for a given neuron. This provided for each neuron the list of areas that it substantially targeted. By grouping neurons based on their common projection lists, we could then highlight the total range of projection motifs that this particular subset of neurons exhibited. From these patterns, we counted the numbers of dedicated inputs to a single-area, as well as bifurcations (i.e., targeting two areas) and trifurcations or higher order motifs (Figure 5F). We found motifs that exhibited first and higher order connectivity reaching up to four jointly targeted somatosensory areas. A preliminary assessment of the statistical significance of VPM projection motifs, which uses the binomial test with Bonferroni correction similarly to how it was achieved in Han et al. (2018), can be seen in Supplementary Figure 1.

We then proceeded to quantifying the distribution of projection motifs in relation to their respective dominant projection types. The areas characterizing each motif were ordered in descending order by the number of received axonal terminals from a single neuron. For instance, motif SSp-bfd - SSs revealed a pattern in which a given neuron most dominantly projected to the somatosensory barrel cortex, while its second most dominant target was the supplemental somatosensory area. We sorted the motifs in descending order by the number of participating neurons to assess the most predominantly targeted areas and we subdivided the motifs in monofocal and multifocal ones to better understand the combinations with which VPM neurons targeted these predominant areas (Figure 5G). A potential issue for multifocal neurons is that their secondary arborizations could be located in the border between the dominant projection area and the secondary one, thus raising concerns about the true nature of such motifs which could be monofocal according to a different anatomical delineation. To address this issue, we further subdivided neurons participating in multifocal motifs in “center” and “border” groups, based on whether they had at least four axonal terminals in the secondary area with a distance >200 μm from the nearest border with the dominant projection area.

#### 2.4.3 Distinguishing morphological gradients and types

**Figure 6:**
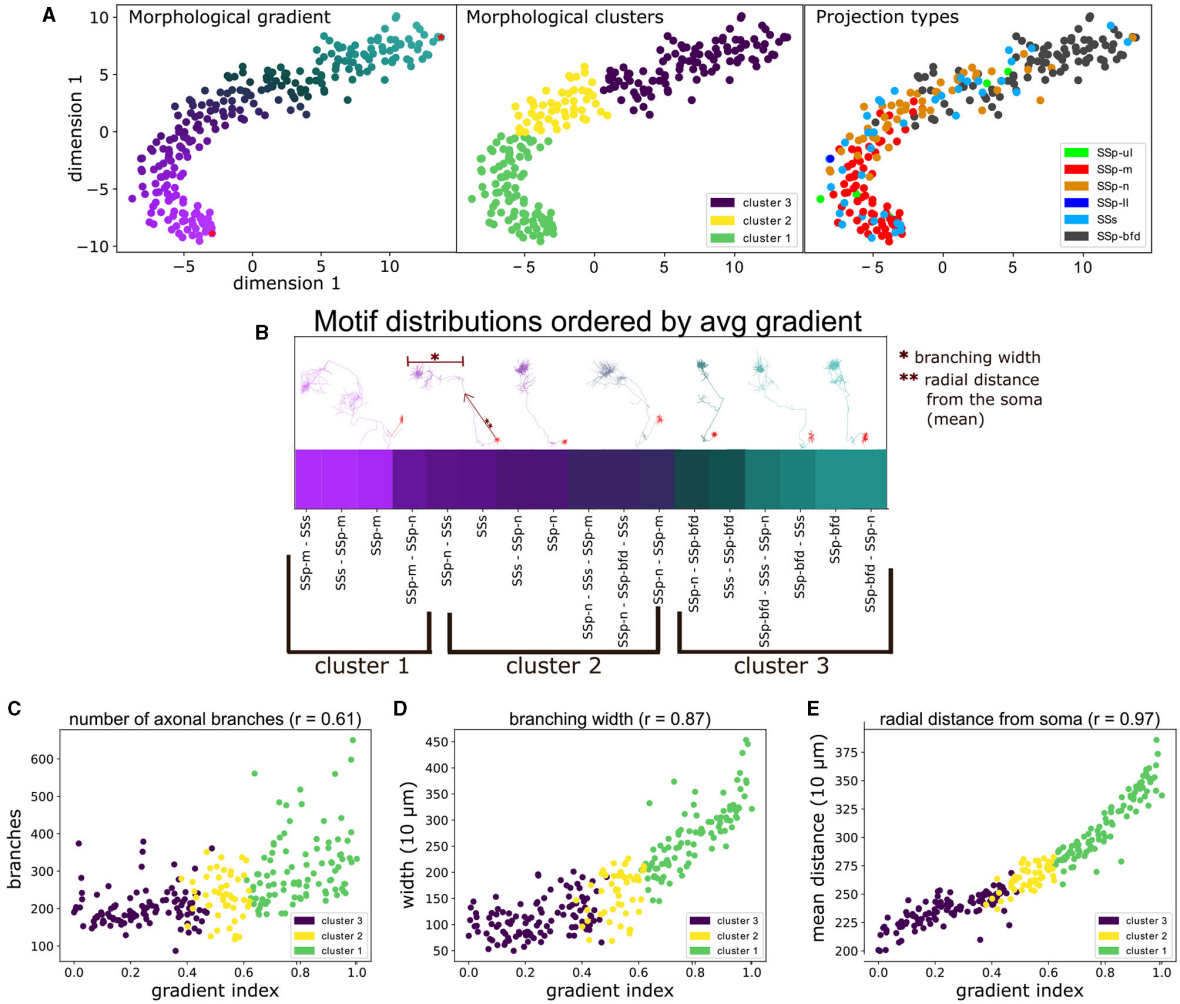
The unbiased characterization of VPM neurons based on their morphological diversity suggests the presence of a gradient that is aligned with their topographical and higher order projection properties, as well as correlated with distinct morphometrical measures. **(A)** Scatter plot illustrating a 2D embedding of the neuronal morphological diversity (see Section 2.4). The plot has been color-coded three times to highlight the similarity between the three predominant approaches used to distinguish cells in this work, namely by: morphological gradient (**A**, left), morphologically-defined type (**A**, middle) and the projection type (**A**, right) of each neuron. The two red dots at the top and bottom right parts of the gradient in (**A**, left) indicate the dorsal-most root point and a ventral anchor point, which we used to normalize the gradient scores in the range 0–1. The legend in (**A**, middle) denotes the color-code of the morphological types, while the legend in (A, right) denotes the color-codes of the projection types, which is described in Figure 4. The *x*- and *y*-axes correspond to the morphological values in the first and second dimension of the 2D embedding space. **(B)** Distribution of the unique projection motifs sorted by the average gradient index of all neurons characterized by each motif (see Section 2.4.2). *x*-axis corresponds to unique projection motifs and *y*-axis corresponds to the average gradient index of neurons that are part of a motif. The labels below the *x*-axis indicate the respective morphological types that characterize the above motifs. The color of each motif corresponds to the color of its respective gradient index as shown in **(A)**. Above the gradient are plotted a number of example neuronal morphologies. Each morphology has the same color as the respective gradient index that sits below its soma position, which highlights the order of the morphology the along the gradient. The arrow next to the second to the right-most morphology and the bar on top of the same morphology are used to exemplify two morphometrics used in this work, the “branching width” and the “radial distance”. The single and double asterisks are used to distinguish the two examples. **(C–E)** Comparison of the gradient indices of VPM neurons with three significantly correlated morphometrical measures (*p* < 0.001), namely number of branching points **(C)**, average branching width **(D)** and average radial distance **(E)**. *x*-axis corresponds to the gradient indices, *y*-axis corresponds to values of the respective measure and the *r* value next to the title of each plot corresponds to the Spearman's *r* correlation coefficient between gradient and morphometric value: 0.61 for branch points, 0.87 for width and 0.97 for radial distance. The color-coding of all scatter plots is consistent with (**A**, middle) and indicates the morphological type that each point (neuron) belongs to.

We subsequently applied the Coherent Point Drift method to delineate morphological clusters using the dissimilarity of their axonal trees as a proximity measure (see Section 2.3 for more details and Figure 6A, left for an example). Prior to CPD registration, both neurons were re-centered such that their soma location is located at coordinates 0,0,0. Given two neurons, we computed their dissimilarity by first finding correspondences between the axonal points of both neurons, followed by applying a rigid registration of the former to the latter neuron. We did not allow the registration algorithm to scale the axonal points, which restricted the transformation to only rotating the target neuron. By eliminating the operations of translation and scaling, we focused on finding the optimal rotation required for one morphology to be registered to another. By determining the mean squared distance after convergence of the registration, we ensured the maximum physical proximity between neurons and focused attention solely to the difference in their axonal branching patterns. That allowed us to decouple morphological difference from the neuronal soma locations or their rotation along the inferior-superior axis.

When CPD was performed over all available morphology pairs, we obtained a dissimilarity matrix between all neurons. This matrix could now be used to characterize the morphological diversity between neurons, which was not possible in the space of complete axonal trees containing a variable number of points across different morphologies. The first step was to reduce the dimensionality of the dissimilarity matrix to its two most dominant dimensions in terms of explaining data variance. As described in Supplementary Section 1.4, we used the t-SNE dimensionality reduction technique to the dissimilarity matrix, which estimates a non-linear embedding of the data that respects the topological distance between data points (van der Maaten and Hinton, 2008). This enabled us to visually inspect the morphological diversity in two dimensions with the use of a scatter-plot. We observed that the highest density of the data was along a 1D ridge, hence the second step was to quantify the observed diversity by defining a morphological gradient that can characterize the data, followed by partitioning the gradient into clusters for a simpler morphological characterization.

To define a morphological gradient implied that we could represent each data point on the two dimensional t-SNE embedding using a single number instead of two coordinates, which was inspired by the representation of the data as a curved line in the scatter plot of Figure 6A. Each neuron was assigned a gradient index based on the geodesic distance between its coordinates and the root point coordinates in the 2D embedding space: the root point was assigned to be the point in the upper right end of the t-SNE embedding, which belonged to a barrel-cortex projecting neuron (Figure 6B). The reason for this selection was that barrel cortex projecting neurons were located in the dorsal part of VPM, hence the higher the gradient index the more ventral the neuron was. This provided a positive correlation in Figures 6C–E (see Section 4 for discussion on the topographical analysis). The gradient indices were normalized by the highest index such that they can be represented in the range 0–1.

We then proceeded to check whether there was an alignment between the morphological gradients and the previously identified projection types or motifs. We overlaid the morphological types with the projection types and assessed their overlap (Figure 6A, right). Therefore, we could now pinpoint potential topographical correlations between the soma locations of each cluster and their respective axonal terminals.

We distributed the unique projection motifs identified in this work by sorting them by the average morphological gradient of the neurons belonging to each motif. Hence, we ordered the motifs along their position in the lower embedding morphological space (Figure 6B) and then assessed whether we could place them within a continuum of morphologies defined by the gradient. We then searched for statistically significant correlations between the gradient indices and a number of morphometrical measures, in order to understand whether the morphological differences assessed using our approach were in agreement with pre-computed morphometrics.

To better understand the morphological diversity, we applied hierarchical clustering to the two dimensional embedding to define three clusters or morphological types (Figure 6A, middle). We thus partitioned the gradient into three topographically organized segments that reflect the dorsal, middle and ventral parts in the coronal plane of VPM that most strongly target the barrel, nose and mouth somatosensory cortices, respectively (Peng et al., 2021) (see Section 3.1 for a discussion on VPM topography). We assessed the distribution of the three morphological types across a number of morphometric measures and we computed which morphometrics exhibit significant differences in their distributions across the three types. The motivation was to understand whether the three morphological types exhibited distinct morphological properties and to highlight such properties, which could help us further interpret the morphological types.

### 2.5 Data visualization

To proceed in visualizing the results, an appropriate data representation was necessary. Initially we represented the projection or morphological types as two binary 4-dimensional arrays in which the first three dimensions corresponded to the three axes of the 10 μm Allen Reference Atlas: 1,320 voxels in the *x*-axis representing the anterior-posterior direction, 800 voxels in the *y*-axis representing the superior-inferior direction and 1,140 voxels in the *z*-axis representing the left-right directions, with the origin being the anterior-superior-left corner of CCF. The fourth dimension corresponded to the number of neuronal types under analysis. The first 4D array was the source array and encoded the soma positions of all neurons and the second 4D array was the target array and encoded the respective axonal terminal positions. For instance, if the target voxel 0,0,0,0 had the value 1, this means that there existed an axonal terminal from the first neuronal type at the point (0,0,0) in the 10 μm PIR orientation. A value of 1 at the corresponding source voxel would likewise reflect the presence of a soma of the first type at this given coordinate.

Moreover, we implemented a color-coding strategy to simultaneously visualize the different neuronal types. Each type received a unique RGB color as its identification color for any subsequent form of visualization. We could now visually distinguish potential topographically organized patterns found across the different neuronal types, by looking at their color distribution in plots of their source and target regions, prior to proceeding to more numerically rigorous analyses.

**Figure 7:**
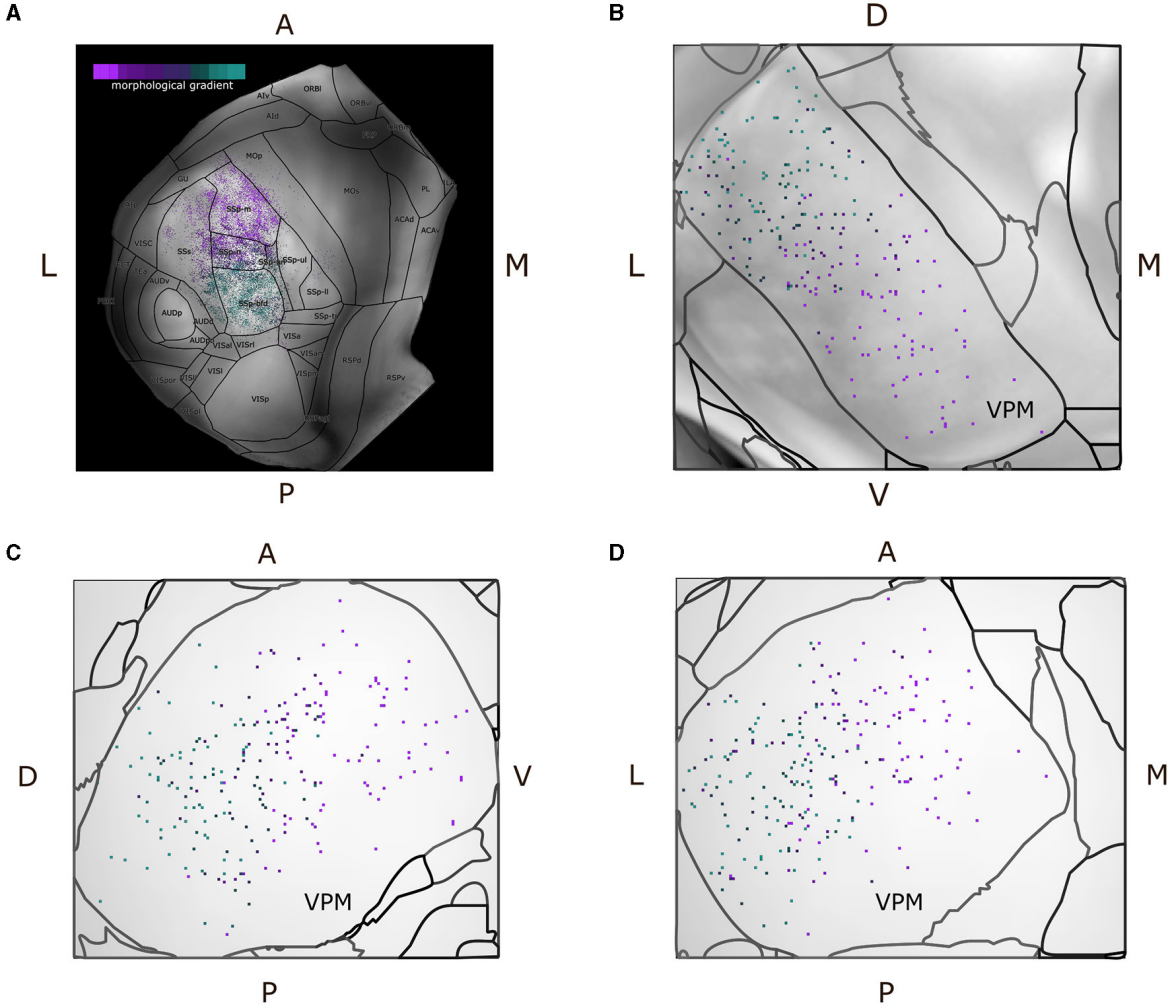
Dorsal cortical flatmap and maximum projection plots of VPM verify the topographical distribution of the morphological gradient that characterizes VPM neurons. **(A)** Dorsal cortical flatmap illustrating the axonal termination patterns of the VPM neurons (13.6 × 13.6 mm along the anterior posterior and left-right axes). The color-coding shown in the top left legend is consistent across panels and is the same used for the morphological gradient index in Figure 6. The correlation between the morphological gradient and the gradient of axonal terminals along the anterior-posterior, lateral-medial axes, as defined by Spearman's *r*, is −0.8, while the equivalent correlation with the gradient of soma positions along the ventral-dorsal, medial-lateral axes is −0.83 (p < 1e-05). The color-coding strategy used consistently throughout figures **(A–D)** is explained in the inset of panel **(A)**. **(B–D)** Visualization of neuronal cell bodies in VPM across coronal **(B)**, sagittal **(C)**, and horizontal **(D)** planes (1.37 × 1.54 × 1.62 mm along the anterior-posterior, superior-inferior and left-right axes, respectively). The letters at the side of each panel denote the plot orientation.

Since we characterized each morphological or projection cluster using different colors, we produced dorsal cortical flatmaps (see Figure 7A) overlaid with anatomical boundaries, as well as 2D projections of VPM (Figures 7B–D, see Supplementary Section 1.2 for the technical implementation details), in order to visually inspect the various clusters. The flatmaps were produced by adapting code from Knox et al. (2018), but we made a number of modifications to improve the anatomical distinction of the projection patterns. In summary, we delineated the anatomical boundaries of ARA using thick black lines, plotted the acronym of each area on its respective center and estimated the flatmap of the STP-based gray matter volumes by taking their average value only across layers 2/3 and 4, such that individual barrels of the barrel cortex become visible. Since the flatmaps are symmetrical and VPM neurons do not have contralateral projections, we created two dorsal flatmaps of only the left hemisphere to reduce memory usage. We therefore mirror the axonal termination patterns of the right hemisphere to the left flatmap one.

To represent the VPM soma position distribution in 2D, three maximum projection plots were created for the coronal, sagittal and horizontal planes, respectively. For each plane, we overlaid the maximum projection of three different components along the plane's axis: first, the color-coded locations of the somata, second, the background gray-matter volume from the STP-images of the CCF v3.0 template (Wang et al., 2020) and third, contour lines that delineated the anatomical borders of VPM according to the ARA parcellation (Figure 7C). Lastly, we imported all the morphologies that were of interest directly to the Scalable Brain Atlas (SBA) composer (Bakker et al., 2015) tool, which enabled an easy-to-use 3D rendering of the data within the CCF template. In SBA, we color-coded the morphologies using the same strategy as described above (see Supplementary Section 1.5).

## 3 Results

Based on the collection of reconstructed neurons collected here we have identified a topographical correlation between the location of source somata in VPM and their arborization patterns in the somatosensory cortices. We analyzed this topographical correlation and identified different neuronal populations inside VPM that preferentially targeted different combinations of somatosensory areas. In the paragraphs below, we will describe these findings in more detail.

### 3.1 VPM neurons can be characterized by their topographical organization, layer target specificity and higher order projections

When embedding in two dimensions the total terminal length per neuron across the somatosensory areas, four of the six areas appeared to be dominantly targeted, namely SSp-m, SSp-n, SSp-bfd and SSs (see Section 2.2 for notations). The data created four sharp clusters, with each cluster being almost completely homogeneous in terms of the dominant projection target of its neuron members; the only exceptions are a few neurons located in the middle of Figure 4A having the SSp-ll and SSp-ul as their dominant projection targets. Therefore, we concluded that neurons could be characterized as dominantly projecting to the primary somatosensory areas representing mouth, nose, whiskers or the supplemental somatosensory area.

When overlaying the projection types onto a dorsal cortical flatmap (Figure 4B), they appeared to be topographically organized. This was evident by the minimal presence of projections outside the dominant projection target, which occurred mostly in the borders between areas, with the exception of the SSp-m neurons targeting the lateral part of SSs. The various somatosensory targets (barrel field, mouth, nose, supplemental) were seen to originate in spatially distinct subareas of VPM. Along the dorsal-ventral and lateral-medial planes inside VPM, neurons appeared to target the barrels, nose and mouth, respectively, which indicated a similar topographical organization of the soma distribution (Figures 4C–E). This finding has been extensively described in the rat VPM (Waite, 1973; Saporta and Kruger, 1977; Ito, 1988; Sugitani et al., 1990) and more recently in the mouse VPM (Peng et al., 2021).

To validate this observation numerically, we fitted for all neurons their soma position to the medoids of their axonal terminal points and extracted the Euler angles describing the rotation required for the former to match the latter, as described in Section 2.4.2. The MSE of transforming the soma coordinates to their target medoids at 10 μm resolution was 931, which we considered adequate (see Figure 4F). The corresponding Euler angles in the *x, y*, and *z* axes, respectively, were −62.8, −1.5, −126.8. We thus deduced that for the VPM somata to match their target centers, they have to perform a negative rotation around the anterior-posterior and lateral-medial axis, with an almost zero rotation around the dorsal-ventral axis. This finding quantitatively validates the above observation that dorsal-ventral and lateral-medial soma positions in VPM correspond to posterior-anterior and lateral-medial axonal terminal locations in the somatosensory cortex.

Regarding preferential laminar targets, a clear majority of VPM neurons projected to layers 2/3 and 4. In SSp-bfd, SSp-n and SSs, layer 4 was the most dominantly targeted layer with 5,546, 5,022 and 3,487 μm of axonal terminal length, respectively, with layer 2/3 being the second most targeted cortical layer (Supplementary Figures 2A, C, D). In SSp-m, layer 2/3 was the most dominantly targeted layer with 3319 μm of axonal terminal length, with layer 4 being the second most targeted cortical layer (Supplementary Figure 2B). When taking into account the layer to which neurons project their highest fraction of axonal terminals length, we identified layer 4 as the selected layer for the SSp-bfd and SSp-n projection-types (53 and 25 neurons, respectively) and layer 2/3 for the SSp-m and SSs projection-types (35 and 23 neurons, see Figures 5A–D). In addition, when assessing the layer with the highest terminal length for the secondary target of multifocal neurons, we identified layer 6b for SSp-bfd and SSp-m (39 and 20 neurons), layer 6a for SSp-n (15 neurons) and layers 4 and 5 for SSs (11 neurons each, Figure 5E).

Regarding interpretation of these findings, projections to layer 4 are well established as the “core” projections that carry feed-forward input from the lemniscal system to the cortex and have been shown to display driver-like characteristics (Sherman, 2016; Harris et al., 2019; Clascá, 2022). Inputs to layer 2/3, although predominantly modulatory, can also evoke the firing of action potentials in some cells, suggesting that both layers participle in the encoding of active touch through direct signals from the thalamus (Crochet et al., 2011; Viaene et al., 2011). Moreover, neurons projecting to layer 2/3 have a shorter length of arbors than the layer 4 projecting ones, as well as shorter projections to the deeper layers, as has been previously reported in Peng et al. (2021). The functional relevance of these secondary projections to the deeper layers is something that has not been experimentally explored in VPM, which has been traditionally considered to be a sensory relay nucleus with point-to-point connections. However, this will be further elaborated in Section 4. Lastly, the secondary projections to layers 2/3 and 4 by multifocal SSs-projecting neurons can be explained by most of these neurons having SSp-m or SSp-bfd as their second most dominantly targeted area, which predominantly receive input in layer 2/3 or 4, respectively, as described above.

Regarding the broadcasting properties of VPM neurons, 26% of VPM neurons were found to be monofocal, with 53% being bifurcating, while smaller fractions of neurons were tri- or quadrifurcating (17 and 1.5%, respectively, see Figure 5F). SSp-m, SSp-n, SSs receive markedly more multifocal projections relative to their monofocal ones (49 multifocal over 20 monofocal projections for SSp-m, 42 over 6 for SSp-n and 34 over 4 for SSs), while SSp-bfd has a more balanced ratio of multifocal to monofocal projections (55 over 38). The presence of monofocal VPM neurons sending dedicated projections to specific somatosensory areas, with individual barrels being the most predominant target, is well known in literature. For instance, in rats it has been reported that 70% of barrel-cortex projecting VPM neurons target individual barrels and 30% targets multiple barrels, with the latter located in the marginal regions of VPM (Sugitani et al., 1990). This raises the question about the presence and function of neurons from first-order nuclei targeting multiple somatosensory areas instead of exclusively targeting a single area (Clascá et al., 2012). To better understand these bifurcated projections, we put the projection motifs within the context of their dominant projection targets and we overlaid them with the morphological gradients, which we discuss in the following paragraphs.

When observing the entire spectrum of VPM projection motifs, 17 out of 40 motifs in total were substantially represented by at least four neurons (Figure 5G). We noticed that monofocal neurons targeted mostly the whisker and the mouth somatosensory areas, while the nose and supplemental areas were represented by only six and four monofocal neurons, respectively. The remaining areas were targeted by multifocal neurons. For each projection type in the barrel cortex and mouth part, the biggest fraction of neurons were monofocal (32 out of 79 and 20 out of 49, respectively), the second largest fraction also targeted SSs (30 and 16 neurons, respectively) and the third largest fraction also targeted SSp-n (12 and 13 neurons, respectively).

To better understand the topographical organization of multifocal projection motifs we assessed the distribution of projections across the primary and secondary projection target areas. For each multifocal neuron, we counted how many terminals were at least 200 μm away from the border between the primary and secondary targets. We used this as a criterion to better understand whether secondary projections were mostly distributed toward the center of the secondary area or more toward the border. Therefore, we classified a multifocal neuron as “center” if it had at least four axonal terminals away from the border between its primary and secondary target areas, or as “border” otherwise. For the majority of multifocal motifs, the number of neurons projecting proximal to the borders of the secondary target was substantially higher than the ones projecting further away from the border (see Figure 5G). The only exception was for joint projections to SSp-m and SSs, for which motif SSs - SSp-m had two border and five center neurons, while motif SSp-m - SSs had eight border and eight center neurons. This suggests that these border-projecting neurons could actually be monofocal ones with broad projections reaching the borders to neighboring areas which could be parts of the dominant projection target in different delineation schemes. This finding will necessitate additional analysis to be carried out in future studies.

Regarding dominant projections to nose and supplemental somatosensory areas, the fraction of secondary projections to the barrel cortex and mouth was almost equal and which shows that the secondary projections are also affected by the topographical organization of VPM neurons across the dorsal-ventral axis (Figure 4B). Within these two projection types there existed two identical trifurcating motifs to the barrel cortex, supplemental and nose, and nose, supplemental and mouth, respectively. However, these motifs never targeted the barrel cortex and mouth together. We thus assume that the barrel cortex and mouth act as two opposite poles in the topographic spectrum of projections and all other projection types exist in between those extremes. This leads to the question of whether there is a morphological difference between neurons participating in these motifs, and between neurons that are monofocal or multifocal.

### 3.2 Morphological diversity of VPM neurons is aligned with their projection and topographical properties

We created a distance matrix over all VPM neurons to better understand their morphological diversity, by deriving the CPD-based morphological distance between all possible pairs (see Supplementary Section 1.4). We represented the morphological diversity by embedding the distance matrix in two dimensions (see Section 2.4), which resulted in a curved-line representation of the data. By aligning this curved line with the dorsal-ventral axis of the VPM soma location, we could now represent the data as a morphological gradient that is further aligned with the other two characterizations of VPM neurons discussed in this work, namely their topography and projection types (Figure 6A, left).

We thus highlighted the relationship between projection and morphological diversity, by overlaying the projection types on top of the gradients (Figure 6A, right), which became more apparent when partitioning of the gradient into three clusters. The clusters could now be interpreted as three zones of projection targets (Figure 6A, middle). Neurons in clusters 1, 2, and 3 primarily targeted either SSp-m, SSp-n and SSp-bfd, respectively, or a fraction of SSs. In each cluster, secondary arborizations were found in either SSs or another primary somatosensory area, with the exception of SSp-bfd and SSp-m never receiving projections from the same neuron.

By ordering all projection motifs in descending order based on their average gradient index of the neurons that belong to them, we put the motifs within the context of the morphological gradient that had a decreasing index along the ventral-dorsal axis (Figure 6B). Cluster 1 involved the ventral-most part of the gradient, in which neurons targeted SSp-m either monofocally or multifocally together with SSp-n and SSs. The transition to cluster 2 involved neurons that targeted SSs and SSp-n, either monofocally or both together and SSp-m or SSp-bfd but never with SSp-bfd and SSp-m being part of the same motif. The transition to cluster 3 involved the dorsal-most part of the gradient, in which neurons targeted SSp-bfd either monofocally or multifocally with SSp-n and SSs. This alignment thus established a straight line of possible somatosensory projections that begins at SSp-m and ends at SSp-bfd and can be seen as a blueprint for rules dictating the projection properties of a random VPM neuron based on the placement of its soma position.

To understand differences in morphologies along the gradient, we correlated the gradient with a number of morphometrical measures and found a statistically significant correlation for three measures, namely mean radial distance from the soma (*r* = 0.97, *p* < 0.001), number of branching points (r = 0.61, p < 0.001) and branching width (*r* = 0.87, *p* < 0.001, see Figure 6B for an example illustration of these morphometrical measures). Therefore, the gradient could be interpreted as a gradual increase in the length, number and width of axonal branches (Figures 6C–E).

We further analyzed the relationship between topography and morphological diversity by overlaying the morphological gradient onto a dorsal cortical flatmap (see Figure 7A). The results were largely in agreement with the topographical organization of projection types described above. However, since the gradient index had a continuous value instead of a discrete category, we could now assess the distribution of the gradient along anatomical space. On the cortical surface, the gradient index was inversely correlated with the axonal terminal positions along the anterior-posterior axis. Within VPM, the gradient index was inversely correlated with the position of the somata along the ventral-dorsal and medial-lateral axes (Figures 7B–D). The reason for inverse correlation was that we selected a neuron with a highly dorsal soma location having the lowest index of the gradient, such that the gradient could exhibit a positive correlation with a number of morphometrics, which we describe in the paragraphs below. The relationship between gradient and topography was further demonstrated when fitting the inferior-superior and right-left coordinate of all VPM soma locations to their respective morphological gradient, which yielded a low MSE of 0.061.

### 3.3 Delineating individual barrels expands the scope of the analysis in the CCF to whisker-specific connectomics

**Figure 8:**
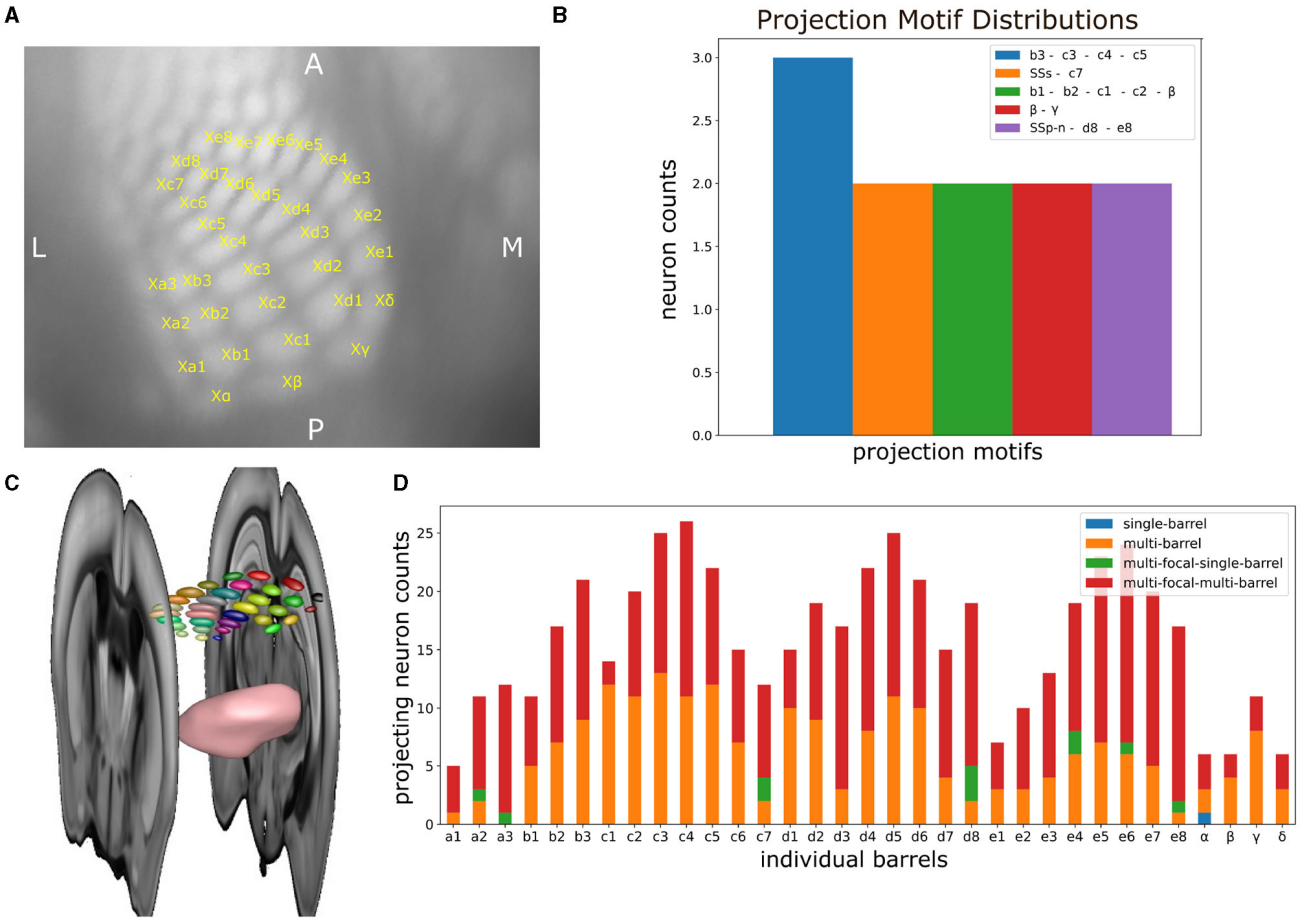
The delineation of individual barrels in mouse barrel cortex enables the systematic analysis of the projection patterns of VPM neurons on a finer-grained level of parcellation than previously possible using the Allen Reference Atlas. **(A)** Inset of a top view cortical flatmap centered around the barrel cortex, in which the distinct individual barrels have been delineated. The name of each barrel is plotted in yellow at the upper right corner of its respective area in the flatmap, while the lower left part of the X character next to each name is used for marking the barrel center. **(B)** Distribution of projection motifs that take individual barrels into account. Motifs are presented in descending order by their distribution of participating neurons. *x*-axis: motif that is color-coded as shown in the legend. *y*-axis: number of neurons participating in a given motif. Name of each motif with each corresponding color in the plot. Motifs with only one participating neuron were not included. **(C)** Snapshot of a 3D rendering of the individual barrels within a whole-brain context. The barrels are rendered as small 3D ellipsoid meshes, with each barrel being visualized with a distinct color. Additional rendered structures include the VPM nucleus as a pink mesh and two consecutive coronal sections in gray-scale marking the boundaries of the barrel cortex along the anterior-posterior axis. **(D)** Distribution of the different projection motifs based on the number of neurons sending at least four axonal terminal projections. *x*-axis: individual barrels. *y*-axis: number of neurons targeting a barrel, which is partitioned in four groups based on the type of their projections. The blue, orange, green, and red colors highlighted in the legend indicate the four different possible projections that a given barrel can receive from VPM neurons, namely projections from monofocal neurons targeting a single barrel (blue), monofocal neurons targeting multiple barrels (orange), multifocal neurons targeting a single barrel (green) and multifocal neurons targeting multiple barrels (red).

We proceeded by applying the same statistical analysis as the one performed in Section 3.1, but now taking into account the individual barrels (Figures 8A, C). See Supplementary Section 1.3 for a detailed description on how the individual barrels were delineated. By assessing the barrel-specific projection motifs, we observe a small number of neurons targeting individual barrels either monofocally or jointly with other barrels or somatosensory areas. From all neurons, only 41 monofocally targeted individual barrels, 30 targeted multiple barrels but no other somatosensory area and 76 targeted a combination of barrels and another somatosensory area. Regarding enriched motifs, we identified only four motifs involving individual barrels that were targeted by more than one neuron. Particularly, three neurons jointly targeted barrels b3, c3, c4, c5, two neurons jointly targeted barrels d8 and e8 and area SSp-n, two neurons jointly targeted barrels γ and β, two neurons jointly targeted barrel c7 and area SSs and two neurons jointly targeted barrels b1, b2, c1, c2, and β (see Figure 8B).

Likewise, when estimating the number of neurons that target each barrel with at least four terminals, we observed that a barrel was targeted on average by 16 neurons, with barrels c4, d5, and c3 being targeted by the highest number of neurons (26, 25, and 25, respectively). When further expanding this analysis by computing the fraction of neurons that are monofocal or multifocal and target single or multiple barrels, we found an overrepresentation of multi-barrel targeting neurons that were either monofocal or multifocal. In particular, only barrel α was targeted by one monofocal neuron in a single-barrel fashion. Barrels a2, a3, c7, d8, e4, e6, e8 were targeted by two multi-focal neurons on average in a single-barrel fashion. The majority of the projections that barrels received originated from multi-focal and multi-barrel neurons (311 projections), followed by multi-barrel and monofocal neurons (201 projections). Taken together, the data suggests that there is additional structure beyond the one barreloid to one barrel projection (see Figure 8D), but the sample size is insufficient to statistically characterize this structure.

## 4 Discussion

In this work, we have developed a workflow for translating ~300 morphological reconstructions of VPM neurons into meso-scale projection statistics. We obtained data from the publicly available repositories of the *MouseLight* (Winnubst et al., 2019) and *Braintell* projects (Peng et al., 2021), which we combined in the analysis to deal with limited sample size. Additionally, we have created a portal for unified access to these morphologies, as well as morphologies from other public databases, called the *Neurons Reunited Portal*, to improve the reusability of morphological data. The statistical analyses were focused on two goals: firstly, analyzing the topographical organization of these neurons in terms of their soma distribution inside VPM and their axonal termination patterns across the different somatosensory areas. Secondly, characterizing the neuronal diversity in terms of their morphological and projection patterns and identifying distinct subpopulations of neurons.

We first used the distribution of terminal branch length of axonal projections across the somatosensory areas as a measure for characterizing neurons by their dominant projection targets. We thus identified different projection motifs, with a fraction of neurons targeting only one area and subsets of neurons targeting two or more areas. For characterizing morphological diversity, we adapted the Coherent Point Drift (CPD) technique (Myronenko and Song, 2010), which uses an Expectation-Maximization strategy to align two point clouds, in this case two morphologies, and then register one cloud to another. We modified CPD to work with axonal trees and used rigid registration to compute the registration error as a distance between all possible morphological pairs. This resulted in a distance matrix which was used to define a morphological gradient characterizing the diversity of all available data, and which we further parcellated into three clusters. We assessed the covariation of the morphological gradient of VPM neurons with their topographical organization and projection motifs by overlaying the motifs on the gradient and aligning the gradient with the dorsal-ventral axis. Regarding their topographical orientation, we aimed to uncover rules to predict where a neuron will terminate its projections in relation to where its soma is located. To accomplish that, we developed 2D visualization tools in the form of dorsal cortical flatmaps and VPM maximum projection plots. This allowed us to place the newly characterized morphologies within the spatial context of the cortical surface and the VPM nucleus while maintaining their diversity in terms of their projection or morphological patterns.

We thus identified a gradient ordering the morphological characterizations of VPM neurons, which was aligned with their topographical organization and with their projection-based characterizations. This suggests non-random morphological differences along the dorsal-ventral axis in VPM, which is in agreement with (Peng et al., 2021), as well as with previous works on the VPM somatotopy of the rat brain (Waite, 1973; Saporta and Kruger, 1977; Ito, 1988; Sugitani et al., 1990). These works have illustrated that VPM soma positions along the ventral—dorsal and medial—lateral axes correspond to axonal arbor positions along the anterior—posterior and lateral—medial axes, which is indicative of a 3D rotation of soma positions relative to their axonal projections. By incorporating neurons from the *MouseLight* repository in addition to the *Braintell* ones, this work has validated this topographical finding of VPM neurons and has extended it with respect to its morphological diversity: neurons can be sorted by a morphological gradient, which is aligned with the dorsal-ventral and lateral-medial axes of their soma positions, and which is characterized by an increase in number of branching points, radial distance from the soma and width of terminal projections. This has implications for spatially resolved models of the thalamus; for a realistic model of VPM, it is not enough to use the above topographical rules for placing an average axonal morphology from the available sample set, but one has to adapt its morphological properties based on where the morphology will be placed.

Before discussing the projection properties of the various morphological characterizations, we first have to acknowledge the importance of previous works in opening up the investigation of the heterogeneity of axonal projections at the individual neuron level. The work of Han and coworkers was instrumental for uncovering the communication patterns between different cortical areas which involve different projection-types of neurons with distinct functional roles in neural circuits (Han et al., 2018). Using neurons from the *MouseLight* database, Morita and coworkers compared the spatial extent of the axonal projection patterns to striatum of intratelencephalic (IT) neurons with pyramidal-tract (PT) neurons and found, despite the intrinsic variability, quantitative differences between those groups (Morita et al., 2019). We extended this line of work to the thalamus and uncovered similar principles as the ones described for the mouse visual neurons: dedicated neurons projecting to one area and broadcasting neurons projecting to multiple brain areas. Hence, information in the thalamus is distributed through ensembles of dedicated or broadcasting pathways with distinct functional implications which is open for investigation in future studies.

These pathways are not homogeneous in space, but as shown above, they can be sorted along the dorsal-ventral axis in VPM in three coarse groups, which are: projections that primarily target the barrel cortex but can also target the nose and supplemental areas, projections that primarily target the nose but can also target the barrel cortex, mouth and supplemental area, and projections that primarily target the mouth but can also target the nose and supplemental area. However, these unique sets of motifs are never targeting the mouth and whiskers together, due to their distance along the dorsal-ventral axis. Therefore, the mouth and whisker representations of the somatosensory cortex act as two opposite poles in the range of VPM projections patterns, between which all above described motifs exist, with the nose representation existing in the middle between the whiskers and the mouth, while the supplemental area is mostly receiving secondary projections. We have thus illustrated how two sources of information, the soma position and the morphological properties of neurons, are spatially correlated with the projection motifs that have been identified in this work.

An interpretation for the function of broadcasting neurons is the encoding of shared information in a form suitable for multimodal associations across subsets of areas (Han et al., 2018). While projections from monofocal neurons are considered to be functionally tailored to their respective target area, projections from multifocal neurons could potentially be related to different functional circuits involving a primary target in layer 2/3 or 4, which in most cases is located in SSp, and secondary targets in the infragranular layers, which in most cases is located in SSs.

One may wonder how these different projection motifs are shaped during brain development. By assessing the morphological properties of the different projection motifs in relation to their topography, we observed a gradual decrease in the length and dispersion of axonal branches and terminals when traversing across the ventral-dorsal axis in VPM. Deriving a causal explanation of this finding is difficult due to the lack of morphological reconstructions across different developmental time-points. We speculate that the reason is related to the anatomical position and the genetic programming of these neurons (Clascá et al., 2012; Clascá et al., 2016; Molnár et al., 2020). An advantage of our CPD-based approach is that it represents a significant extension of existing tools; it does not require anatomical labels to characterize neurons. It is unbiased and takes into account only the axonal tree, but it does so to the fullest. Moreover, the registration of neurons to CCF allows them to be integrated with other similarly registered datasets and to be cross-validated by other characterizations of neurons based on their electrophysiological (Gouwens et al., 2020), transcriptomic (Lein et al., 2007), or cell density (Kim et al., 2017) properties, for instance.

Despite VPM being selected as a suitable use-case, the workflow can be extended to any thalamocortical circuit of interest provided that an adequate number of reconstructed morphologies is available. While there is no clear definition of an adequate sample size, the coverage of all topographical subdivisions of a thalamic nucleus using LRPN morphologies necessitates the reconstruction of hundreds of individual neurons. This is currently only the case for VPM; the second, third and fourth most densely sampled nuclei are the ventral anterior-lateral complex of the thalamus (VAL) with 41 neurons, the ventral posterolateral nucleus of the thalamus (VPL) with 36 neurons, and the posterior complex of the thalamus (PO) and the mediodorsal nucleus of thalamus (MD) with 17 neurons each.

Previous works have registered thalamocortical neurons of the rat in local volumetric structures representing the barrel cortex (Egger et al., 2014; Udvary et al., 2022). A major advantage of our approach compared to these works is that our analysis can be applied to morphologies that have been registered to the whole-brain template of CCF and is thus not restricted to well-defined but localized models of individual barrels. While one can obtain insights regarding intra-barrel connectivity with such localized approaches, directly connected functionally relevant structures such as other somatosensory areas and thalamic nuclei are not being explicitly modeled. As a consequence, the topographical organization and higher order connectivity of these neurons is lacking. By modeling these interactions on a whole-brain scale, one can directly use this information from the morphological reconstructions without approximation.

We would like to discuss a number of issues that we encountered when implementing this analysis workflow. Primarily, aligning thalamic neurons with each other or with the pial surface is a tough problem given their curvature at the sub-cortical level and the overdispersion of terminal trees at the cortical level. This makes it challenging to create consensus neurons that can be considered examples of each projection or morphological type, similarly to how it was done in Gao et al. (2022). Another challenging issue is finding accurate point-to-point correspondences between two axonal trees similarly to what has been done successfully in dendrites (Batabyal et al., 2020). This is due to the presence of hundreds of terminals and hundreds of branches, which results in a computationally demanding task for dynamic programming algorithms that could potentially be more accurate than CPD.

Additionally, 26 out of 256 *Braintell* neurons have serious registration issues (see Section 2.2 for more details), which renders them inappropriate for being incorporated in this analysis. Furthermore, we currently do not have densely enough sampled morphologies to estimate statistically significant axo-dendritic appositions and we do not have registered interneurons in order to derive a proper inhibitory to excitatory ratio of projections (Liu et al., 2023). Lastly, information about pre-synaptic boutons is currently not available in both the *MouseLight* and *Braintell* datasets and as such the workflow cannot incorporate more elaborate analyses involving the VPM bouton distribution as the ones shown in Casas-Torremocha et al. (2019), Rodriguez-Moreno et al. (2020), and Casas-Torremocha et al. (2022). We here highlight these issues by emphasizing them as worthy problems to tackle.

As a consequence, we suggest to turn these issues into an open call to the neuroscience community to work synergetically together using portals such as the one presented here to create unifying scaffolding models underlying the network connectivity of the mouse brain and potentially extending to other species. We thus release this pilot study, which for the points addressed above could serve as a valuable resource to the community, whose feedback and experiences we would like to use to improve our efforts. This work does not develop new anatomical techniques, data analysis algorithms, or simulation approaches. The existing ones are adequate, but nevertheless have failed to fulfill expectations because they were used in isolation. Single-cell labeling methods, combined with cutting edge neuroinformatics tools are utilized here together within a collaborative infrastructure. The innovation is thus to link together the different approaches using model-based analysis. In the following paragraphs we propose a number of future extensions to this work that could be beneficial to the community.

The current analytic workflow will be updated to incorporate reconstructed neurons from a small repository of in-house manually traced and precisely placed neurons from a recently developed pipeline whose description is in a work currently under preparation. Besides incorporating more data into the analysis, the reason for including this repository is to use CPD for estimating one-to-one matches between the in-house neurons and the current *MouseLight* and *Braintell* morphologies. Thus, we intend to reformulate our approach as a curation tool for validating the registration quality of neuronal morphologies. This matching can then lead to a thorough assessment of registration accuracy in terms of coverage of cortical layers and subcortical nuclei, in which the in-house neurons will act as the ground truth given their curation by neuroanatomical experts. By creating a 2D embedding of the in-house morphological diversity in comparison to the publicly available large datasets, one can interpret the presence of any identified deviations in diversity. Failure to properly account for such deviations could be considered to be the consequence of a misregistration. Potential divergences between the two datasets can be mitigated by registering neurons to the in-house ones. Hence, this approach can make our workflow applicable to neurons reconstructed in smaller laboratories.

As an additional step, we intend to characterize the overlap between axonal and dendritic arbors for cell-type-specific connectivity motif characterization, which can be achieved by registering interneurons from the *Neuromorpho* database (Ascoli et al., 2007) and incorporating them in our analysis. This is a necessary step for cell-type-specific connectivity estimation since the current analysis takes only LRPN neurons into account and omits other cell-types such as PV, VIP or SST inhibitory cells. The connection between two cells would be inferred by applying touch detection algorithms between the axonal arbors and dendritic spines of distinct cells, in a similar fashion to Egger et al. (2014) and Udvary et al. (2022). However, this approach requires sufficient data to form a dense representation of cells within a particular brain area. That said, there are too few single-neuron morphologies for densely filling the volumetric space in the same way that was achieved by tract-tracing experiments (Oh et al., 2014; Harris et al., 2019); hundreds of more reconstructions would be required for such an ambitious task. We thus intend to replicate the axonal trees of current LRPN neurons based on rules derived by observing the much denser tract-tracing experiments of the Allen Mouse Brain Connectivity Atlas (van Albada et al., 2022).

While we focus on single-neuron axonal reconstructions in mice, we also acknowledge the development of promising new directions in single-cell connectomics. Recent advances, referred to as the semi-automated reconstruction and tracing (SMART) technique have made it possible to image axonal branching patterns from a few simultaneously labeled long-range projection cells in the mediodorsal nucleus of the thalamus in the non-human primate (Xu et al., 2021). Interestingly, also more diverse, non monofocal projections were observed in this dataset. Moreover, the axonal morphologies contain details at resolutions below 100 nm that are often not visible in the *Braintell* and *MouseLight* databases. The newly developed *ExA-SPIM* technology (Glaser et al., 2023) can by expanding brains reach this limit at the whole brain scale, and furthermore, scan axonal morphologies without having to resort to sectioning, which would have made segmentation and tracing approaches challenging. This technology promises a vastly larger yield of fully reconstructed neurons.

Taken together, we have developed a novel paradigm for addressing three challenging questions in single-cell connectomics. First, translating axonal morphologies into meso-scale projection statistics that are capturing previously not accessible higher order connectivity patterns. Second, characterizing their morphological diversity by first computing the morphological distance between neurons with a probabilistic approach, followed by a lower dimensional embedding which takes the distance into account and is not dependent on pre-computed morphometrics. Third, assessing the topographical correlation between the distribution of their somata and respective axonal terminals. By using VPM as an ideal use-case, we have identified a gradient in the lower dimensional embedding which aligned their topographical organization along the dorsal-ventral and lateral-medial axes with an increase in their number of terminals, length and width of branches, and a number of projection motifs along the anterior-posterior axis that involved a spectrum of outputs from the primary somatosensory mouth to the supplemental area, nose, and then to the barrel cortex, with different combinations of monofocal dedicated output to a single area and multifocal output to two-to-three combinations of the above areas. Lastly, we have served the community with a unified access portal for visualizing in 3D fully reconstructed neurons on the order of 10,000. The portal is connected with scripts written in the Python programming language. for the above mentioned analysis, as well as with 2D visualization tools in the form of cortical flatmaps and subcortical maximum projection plots. To support open science, the workflow will be uploaded on Github and become part of the EBRAINS infrastructure. This work opens the way for a collaborative analysis framework in single-cell connectomics, which can unify the data-driven topographical, morphological and projectome analysis derived from long-range projection neurons. By turning the framework into predicting properties of previously unseen neurons, the community can generate new hypotheses on structural neuronal networks to be validated with electrophysiological, tracing or optogenetics experiments.

## 5 Information sharing statement

The analysis workflow described in this work has been designed and tested in the form of a Jupyter Notebook together with a number of supporting libraries in Python, which have been published online with their description at the EBRAINS Collaboratory and at Github. In addition, this work inspired the creation of two neuroinformatics-related tools, namely *mesoscale-extractor* and *neuron-aligner*. From the two tools, *mesoscale-extractor* extracts mesoscale connectivity statistics from a subset of user-selected morphologies of single-neurons, and *neuron-aligner* uses the CPD tool to search for morphologies similar to a morphology selected by the user. See Table 1 for links to the public repositories of the tools and modules mentioned here, as well as for the *Neurons Reunited Portal* database viewer.

## Data availability statement

The datasets presented in this study can be found in online repositories. The names of the repository/repositories and accession number(s) can be found in the article/Supplementary material.

## Author contributions

NT: Writing—original draft, Writing—review & editing. RB: Data curation, Formal analysis, Investigation, Methodology, Software, Writing—review & editing. MR-T: Formal analysis, Investigation, Validation, Writing—review & editing. CA-M: Writing—review & editing. MG-A: Writing—review & editing. FC: Funding acquisition, Writing—review & editing. PT: Conceptualization, Funding acquisition, Supervision, Validation, Writing—review & editing.
