# Supplementary material for: Translating single-neuron axonal reconstructions into meso-scale connectivity statistics in the mouse somatosensory thalamus
Source: Front Neuroinform. 2023 Dec 1;17:1272243. doi: 10.3389/fninf.2023.1272243 (PMC10722239; doi:10.3389/fninf.2023.1272243)
Supplement: Supplementary file 1 [file Data_Sheet_1.PDF]

Supplementary Material

1 SUPPLEMENTARY METHODS

1.1 Data repositories

The *MouseLight* database is comprised of $\sim$ 1500 complete morphological reconstructions of single neurons (Economo et al., 2016). These neurons are primarily located in the thalamus, motor cortex, subiculum and hypothalamus and their morphologies were reconstructed using a high-throughput procedure involving viral projection labeling, high resolution imaging and anatomical segmentation. They first labeled single neurons by injecting 56-days-old (P56) male and female wild-type mice with two adeno-associated viruses (AAV): low-titer AAV expressing cre-recombinase (AAV Syn-iCre) and high-titer AAV coding for a green fluorescent protein (AAV CAG-Flex eGFP/tdTomato). To produce high-resolution images containing the labeled neurites, they used serial two-photon tomography with an integrated vibratome. For tracing and reconstructing the neurons, they implemented a semi-automated algorithm for the segmentation and reconstruction of the soma, axons and dendrites that required manual intervention for distinguishing branches at intersection points. A prime example was the capability to identify cell-types with hitherto unknown projection patterns, such as in the case of zona incerta and subiculum neurons (Winnubst et al., 2019).

The *Braintell* database comprises of $\sim$ 1700 fully reconstructed single-neuron morphologies from cortex, claustrum, thalamus, and striatum (Peng et al., 2021). This repository is a product of a pipeline designed for labeling, imaging, reconstructing, registering and analyzing single neurons from these areas. To label single cells, they used a combination of two mouse transgenic lines, namely GFP-expressing Ai139 or Ai140 TIGRE2.0 reporter and the TIGRE-MORF reporter (Madisen et al., 2015; Daigle et al., 2018; Veldman et al., 2020). This leads to labeling of a sparse number of cells, but for each the complete axonal and dendritic arborization is labeled. They performed anterograde tracing on $\sim$ 140 P56 male and female transgenic mice. The brains were imaged using the fluorescence micro-optical sectioning tomography (fMOST) imaging platform (Li et al., 2010), which integrates epifluorescence microscopy with a system for mechanical sectioning. For each 3D imaged brain, they automatically reconstructed the underlying neuronal morphologies using the Vaa3D open source software (Peng et al., 2014), which were then registered to CCF with the mBrainAligner tool (Peng et al., 2011).

They characterized the morphological diversity of the above described areas and identified cell-types that could be distinguished by their projection patterns. By analyzing the diversity of these projection types, they found that it was correlated by multiple factors including transcriptomic composition, convergence of projections, laminar-specificity for cortical neurons and topographical organization.

For both repositories, the mice were housed in an enriched environment with multiple litter mates. However, *MouseLight* matched litter mates by sex, whereas *Braintell* did not control for sex. The degree of enrichment and gender could have an effect on the morphologies, however, a control analysis showed that the VPM morphologies as a population had similar properties.

1.2 Flatmap visualizations

Harris et al. developed cortical flatmaps for visualizing in 2D of neuroanatomical data along the cortical surface (Harris et al., 2019). They followed the two-step procedure according to which they defined a curved cortical coordinate system and then smoothly mapped the cortical surface to a flatmap. For defining

the curved coordinate system, they represented the cortical surface in three dimensions, with the first two representing the anterior-posterior and right-left axes and the third representing streamlines which matched columnar cortical structures and quantified cortical depth. They computed the streamlines by using the Laplace equation (Griffiths, 2013) to estimate the orthogonal path between the white matter and the pia surface. By averaging streamlines over a specified range of cortical depth, one can then obtain a 2D projection of the data.

For creating the flatmap, they computed the geodesic distance between each surface point and two anchor points representing the two axes of the 2D flatmap embedding. The respective the flatmap position of each cortical surface point was estimated as the one for which the radial distance from the anchor points in 2D was equal to the geodesic distance between the surface point and the anchors in 3D.

1.3 Segmentation of the barrel cortex

The primary whisker somatosensory cortex or else known as barrel cortex (SSp-bfd) is involved in specialized processing of sensory information that is received from the mystacial vibrissae (Brecht, 2007; Diamond et al., 2008; Petersen, 2019; Staiger and Petersen, 2021). The barrel cortex is comprised of individual distinct barrels in layer 4, which are horizontally distributed along the anterior-posterior axis. Individual whiskers are mapped onto individual barrels resulting in a somatotopic organization with specialized functional information processing pathways (Woolsey and Van der Loos, 1970).

When averaging the STP-intensity volumes representing the gray matter contrast of 1675 brains to develop CCF v3.0 (Wang, 2020), individual barrels of the mouse whisker barrel fields became visible in layer 4. We validated this when constructing the cortical flatmap visualizations (see Main fig. 7 A for an example), because the flatmap intensity was computed by taking the maximum value in the 3D intensity volume only across layers 2/3 and 4.

This finding encouraged us to further proceed in characterizing projection and morphological patterns with respect to individual barrels and rather than considering the barrel cortex as a single anatomical module. We thus proceeded to label the individual barrels in the flatmaps, infer their positions in the original 3D volumes, expand these putative barrels to include their respective cortical columns along the entire cortical depth, and lastly analyze barrel-specific projection motifs.

For labeling individual barrels, we relied on the help of expert neuroanatomists. We created a png image of the dorsal cortical flatmap, and asked the anatomists to mark the center of each individual barrel with its respective name using the Inkscape software package (Kirsanov, 2009) (see Main fig. 8 A). We then loaded the images in a Python script and estimated the 2D coordinates of each labeled barrel center using the xml minidom library, which is a Python implementation of the Document Object Model interface (see Main table 1).

We then used the flatmap streamlines to infer the respective positions of each barrel center in the 3D volume. Given the coordinates of each barrel center, we selected their respective streamline along the cortical depth and estimated the middle voxel across the ones belonging to layer 4. We considered these barrel center points as seed points for delineating the remaining volume of each barrel as follows.

With the seed as the initial point of a given barrel, we first estimated the maximum radius that a sphere centered around that point should have, by taking the euclidean distance between its coordinates and those of its nearest neighboring seed point. We then specified two exemplary intensity values, which we termed core and ring. Core corresponded to points with a distance from the seed less than 10%, and ring corresponded to points with a distance from the seed between 45% and 55%, with 100% being equal to the maximum radius.

We then utilized an iterative algorithm for expanding the borders of the barrel until they matched the

borders shown in the flatmap. The original borders were defined by all core points of the barrel. For every step, the borders were expanded by being dilated with a sphere having a 10 μm radius. For all new points added to the volume after dilation, we discarded those having an intensity below a threshold level. The threshold level was defined as the average intensity between the core and the ring intensity. The algorithm halted when no new point with intensity exceeding the threshold could be added in the barrel volume (see Main fig. 8 C).

Furthermore, we constructed an expanded version of each barrel that would correspond to a full cortical column. This was achieved by assigning the label of each barrel to all the voxels belonging to the same streamline as the voxels that have already been labeled as part of the barrel. In essence, this operation was an expansion of each barrel labeling along the cortical depth axis based on the streamlines computed by (Knox et al., 2018) for including all layers beyond 2/3 and 4.

1.4 Dimensionality reduction and Clustering

Given that the neuronal morphologies are comprised of a variable number of points each, this makes statistical comparisons challenging. To account for that, we create a lower dimensional embedding of the neurons, in order to visualize and quantify their morphological diversity.

How can one reduce the dimensions of a dataset, when its data points do not have the same number of features, which in this case is the number of axonal points? The solution is given by CPD which, as shown in Main section 2.3, allows us to compute the morphological dissimilarity between morphologies. This similarity matrix can now be provided to any dimensionality reduction technique as an input, since it is in most cases a pre-processing step prior to the reduction of dimensions. For a visually intuitive approach of analyzing this morphological dissimilarity, we selected the t-stochastic neighbor embedding (t-SNE) non-linear dimensionality algorithm (van der Maaten and Hinton, 2008).

The non-linear embedding estimated by t-SNE makes it optimal for visualizing the data in two or three dimensions in a manner that respects the topological properties of the data. This means that morphologies that were similar in the space of the actual data will also be similar in the low dimensional space. We embedded the data in two dimensions, given that two dimensions were sufficient in accurately representing the proximity in the original space. This was evident by a low Kullback-Leibler divergence value of 0.38, as well as when visualizing the distribution of the projection clusters in the two dimensions which were not randomly distributed (see Main section 2.4).

We created scatter plots to illustrate the relationship between the data in the two or three embedded dimensions. Scatter plots are the most frequent visualization tool for t-SNE plots, since by using the x- and y-axes to represent the embedded dimensions, one can shed light on the distribution of data points and their proximity, as well as indicate the presence or absence of distinct clusters of data.

Instead of the presence of clusters, the t-SNE scatter plot suggested the presence of a morphological gradient (Main fig. 7 E), because of the appearance of the data as a continuous curved line. See Main section 2.4 for details regarding how we quantified this gradient and correlated it with the projection motifs and morphometrical measures of the neurons analyzed in this study. Lastly, we used hierarchical clustering to partition the gradient into three distinct morphological clusters. This was done to better understand if the various projection-type neurons we have uncovered in this analysis are non-randomly distributed along the gradient. Hierarchical clustering is a popular clustering approach that can be used to infer a hierarchy between all data points and their respective clusters, which can be visualized as a dendrogram. A dendrogram representation can offer an improved interpretability of the result when compared to parametric clustering methods (Day and Edelsbrunner, 1984), since it encodes the relationship between data points not

being part of the same cluster. Furthermore, neurons can be color-coded based on their clusters to reflect their morphological similarity, which can then be overlaid on top of the t-SNE scatter plot to make the cluster delineation more visually discernible.

1.5 SBA Composer

The Scalable Brain Atlas (SBA) Composer is a web-based application for displaying brain imaging, volumetric and 3d-object-based data embedded within a number of available brain atlases (Bakker et al., 2015). It can be used directly by users or by third party websites as a visualization front-end (see Main table 1). It uses the anatomical parcellation of the Allen Brain Atlas, the areas of which can be rendered alongside the imported data with parameters for color and transparency that can be selected by the user. Moreover, the user can use a computer keyboard and mouse to click and navigate by rotating, scaling and translating the rendered brain objects along the generated scene. Each registered morphology is integrated to SBA by representing connections between the coordinates of its axonal segments as lines. The morphological data can be stored in the Extensible 3D (x3d) file format and can be sent to SBA via an Application Programming Interface.

2 SUPPLEMENTARY TABLES AND FIGURES

2.1 Figures

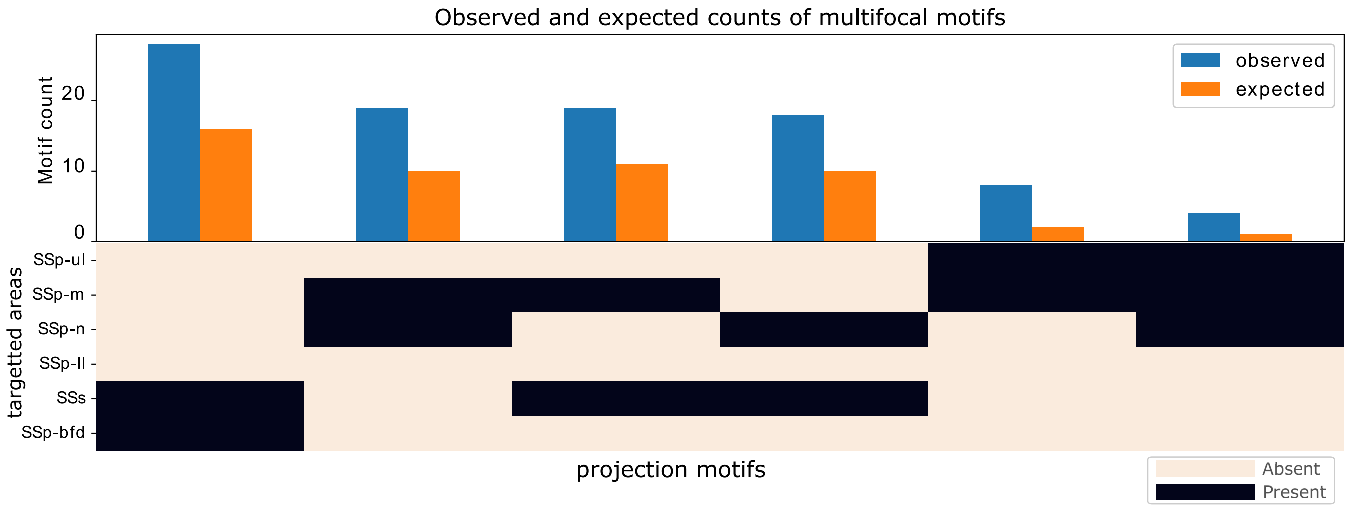

Figure S1. Assessing the statistical significance of the uncovered projection motifs. The significance in over- or under-representation of all multifocal motifs, based on differences between their observed and expected enrichment in neurons, was tested using the binomial test with Bonferroni correction. The upper panel shows the differences in enrichment between observed (blue) vs expected (orange) motifs, with each column corresponding to a motif. The bottom panel is intentionally aligned with the top one, since its column contains the same motif as above, and illustrates the motifs in a 'checkerboard'-like fashion. All available somatosensory areas are shown to the left of the rows, while the black and white colors correspond to the presence or absence of a projection by a neuron, respectively. In the first column for instance, a number of neurons jointly target the barrel field and the supplemental somatosensory area but not the other areas. The test was originally performed in visual cortex neurons in (Han et al., 2018). The expected enrichment is the null hypothesis of the test and assumes statistical independence in the projections of a neuron targeting multiple brain areas. The expected independence value is thus computed by taking the product between the probabilities of targeting each individual area. In this plot, only significantly over- or under-represented motifs have been plotted. Motifs with less than four neurons participating were not included.

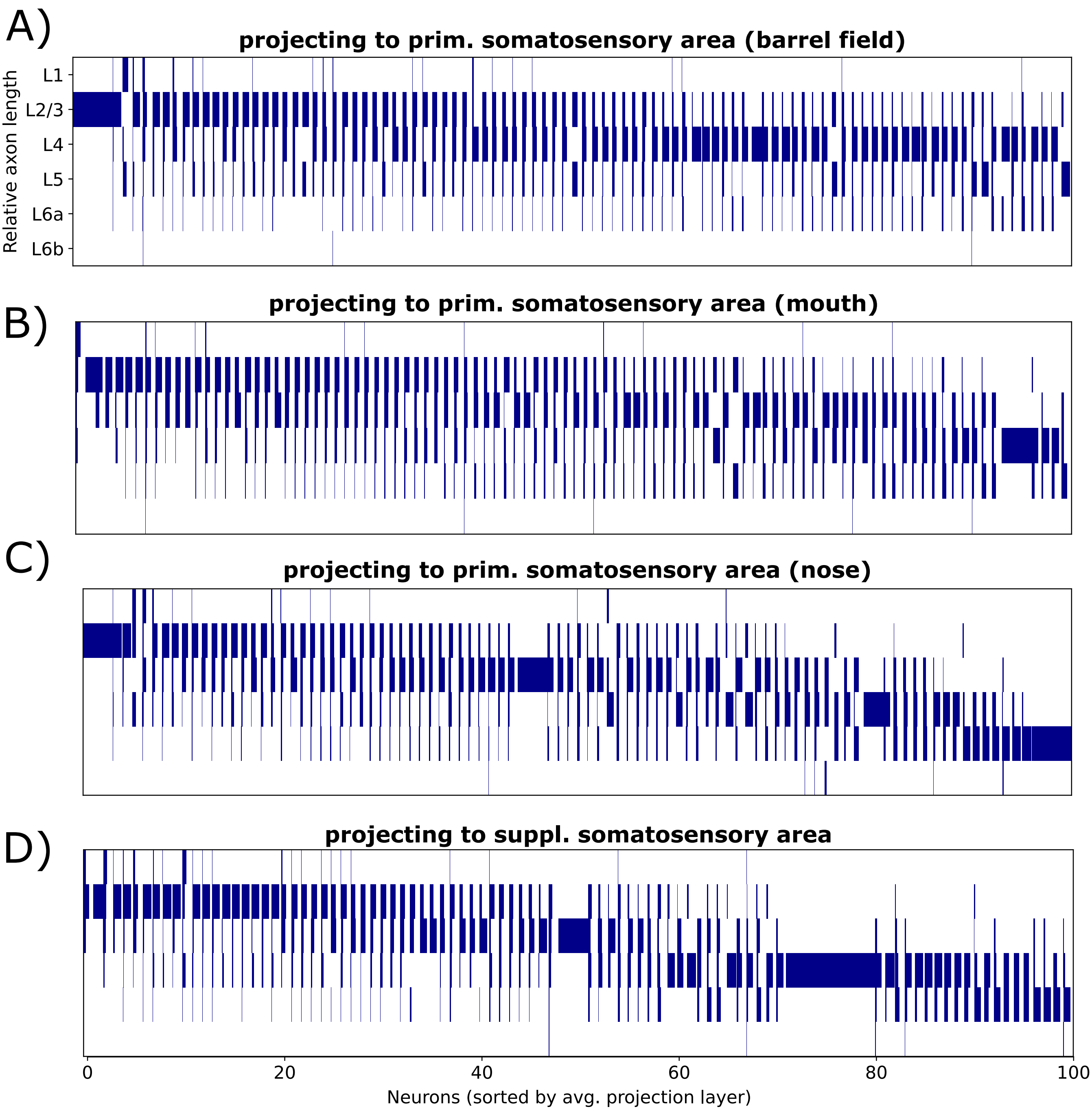

Figure S2. Four plots highlighting the distribution of the axonal terminal branch length of each VPM neuron across all cortical layers expressed in μm that terminates in the barrel field (A), mouth (B), nose (C) and supplemental (D) somatosensory areas. x-axis: cortical layers. y-axis: index of a neuron sorted by its most strongly targeted layer in the descending order of laminar depth from 2/3 to 6b, since layer 1 is weakly targeted. Each blue vertical bar corresponds to the axonal length of a neuron at a given layer that is normalized across all layers. The horizontal width of a blue bar is proportional to the normalized terminal length and the small wide horizontal gap between the bars represents the maximum width of each bar. For each somatosensory area, only the first 100 neurons with the largest length of axonal terminal branches in that area are plotted to emphasize the most dominant projections.

REFERENCES

Bakker, R., Tiesinga, P., and Kötter, R. (2015). The scalable brain atlas: Instant web-based access to public brain atlases and related content. *Neuroinformatics*, 13(3):353:366.

Brecht, M. (2007). Barrel cortex and whisker-mediated behaviors. *Current Opinion in Neurobiology*, 17(4):408–416. Sensory systems.

Daigle, T. L., Madisen, L., Hage, T. A., Valley, M. T., Knoblich, U., Larsen, R. S., Takeno, M. M., Huang, L., Gu, H., Larsen, R., Mills, M., Bosma-Moody, A., Siverts, L. A., Walker, M., Graybuck, L. T., Yao, Z., Fong, O., Nguyen, T. N., Garren, E., Lenz, G. H., Chavarha, M., Pendergraft, J., Harrington, J., Hirokawa, K. E., Harris, J. A., Nicovich, P. R., McGraw, M. J., Ollerenshaw, D. R., Smith, K. A., Baker, C. A., Ting, J. T., Sunkin, S. M., Lecoq, J., Lin, M. Z., Boyden, E. S., Murphy, G. J., da Costa, N. M., Waters, J., Li, L., Tasic, B., and Zeng, H. (2018). A suite of transgenic driver and reporter mouse lines with enhanced brain-cell-type targeting and functionality. *Cell*, 174(2):465–480.e22.

Day, W. H. E. and Edelsbrunner, H. (1984). Efficient algorithms for agglomerative hierarchical clustering methods. *Journal of Classification*, 1(1):7–24.

Diamond, M. E., von Heimendahl, M., Knutsen, P. M., Kleinfeld, D., and Ahissar, E. (2008). 'where' and 'what' in the whisker sensorimotor system. *Nature Reviews Neuroscience*, 9(8):601–612.

Economo, M. N., Clack, N. G., Lavis, L. D., Gerfen, C. R., Svoboda, K., Myers, E. W., and Chandrashekar, J. (2016). A platform for brain-wide imaging and reconstruction of individual neurons. *eLife*, 5:e10566.

Griffiths, D. J. (2013). *Introduction to electrodynamics*. Pearson, Boston.

Han, Y., Kebschull, J., Campbell, R., Cowan, D., Imhof, F., Zador, A. M., and Mrsic-Flogel, T. D. (2018). The logic of single-cell projections from visual cortex. *Nature*, 556(5):51–56.

Harris, J. A. et al. (2019). Hierarchical organization of cortical and thalamic connectivity. *Nature*, 575:195–202.

Kirsanov, D. (2009). *The Book of Inkscape: The Definitive Guide to the Free Graphics Editor*. No Starch Press, USA, 1st edition.

Knox, J. E., Harris, K. D., Graddis, N., and Whitesell, J. D. (2018). High resolution data-driven model of the mouse connectome. network neuroscience. *Neuroscience*, 3(1):217–236.

Li, A., Gong, H., Zhang, B., Wang, Q., Yan, C., Wu, J., Liu, Q., Zeng, S., and Luo, Q. (2010). Micro-optical sectioning tomography to obtain a high-resolution atlas of the mouse brain. *Science*, 330(6009):1404–1408.

Madisen, L., Garner, A. R., Shimaoka, D., Chuong, A. S., Klapoetke, N. C., Li, L., van der Bourg, A., Niino, Y., Egolf, L., Monetti, C., Gu, H., Mills, M., Cheng, A., Tasic, B., Nguyen, T. N., Sunkin, S. M., Benucci, A., Nagy, A., Miyawaki, A., Helmchen, F., Empson, R. M., Knöpfel, T., Boyden, E. S., Reid, R. C., Carandini, M., and Zeng, H. (2015). Transgenic mice for intersectional targeting of neural sensors and effectors with high specificity and performance. *Neuron*, 85(5):942–958.

Peng, H., Bria, A., Zhou, Z., Iannello, G., and Long, F. (2014). Extensible visualization and analysis for multidimensional images using vaa3d. *Nature Protocols*, 9(1):193–208.

Peng, H., Chung, P., Long, F., Qu, L., Jenett, A., Seeds, A. M., Myers, E. W., and Simpson, J. H. (2011). Brainaligner: 3d registration atlases of drosophila brains. *Nature Methods*, 8(6):493–498.

Peng, H., Xie, P., Liu, L., Kuang, X., Wang, Y., Qu, L., Gong, H., Jiang, S., Li, A., Ruan, Z., Ding, L., Yao, Z., Chen, C., Chen, M., Daigle, T. L., Dalley, R., Ding, Z., Duan, Y., Feiner, A., He, P., Hill, C., Hirokawa, K. E., Hong, G., Huang, L., Kebede, S., Kuo, H.-C., Larsen, R., Lesnar, P., Li, L., Li, Q., Li, X., Li, Y., Li, Y., Liu, A., Lu, D., Mok, S., Ng, L., Nguyen, T. N., Ouyang, Q., Pan, J., Shen, E., Song, Y., Sunkin, S. M., Tasic, B., Veldman, M. B., Wakeman, W., Wan, W., Wang, P., Wang, Q., Wang, T., Wang, Y., Xiong, F., Xiong, W., Xu, W., Ye, M., Yin, L., Yu, Y., Yuan, J., Yuan, J., Yun, Z., Zeng, S.,

Zhang, S., Zhao, S., Zhao, Z., Zhou, Z., Huang, Z. J., Esposito, L., Hawrylycz, M. J., Sorensen, S. A., Yang, X. W., Zheng, Y., Gu, Z., Xie, W., Koch, C., Luo, Q., Harris, J. A., Wang, Y., and Zeng, H. (2021). Morphological diversity of single neurons in molecularly defined cell types. *Nature*, 598(7879):174–181.

Petersen, C. C. H. (2019). Sensorimotor processing in the rodent barrel cortex. *Nature Reviews Neuroscience*, 20(9):533–546.

Staiger, J. F. and Petersen, C. C. H. (2021). Neuronal circuits in barrel cortex for whisker sensory perception. *Physiological Reviews*, 101(1):353–415. PMID: 32816652.

van der Maaten, L. and Hinton, G. (2008). Visualizing data using t-sne. *Journal of machine learning research*, 9:2579–2605.

Veldman, M. B., Park, C. S., Eyermann, C. M., Zhang, J. Y., Zuniga-Sanchez, E., Hirano, A. A., Daigle, T. L., Foster, N. N., Zhu, M., Langfelder, P., Lopez, I. A., Brecha, N. C., Zipursky, S. L., Zeng, H., Dong, H.-W., and Yang, X. W. (2020). Brainwide genetic sparse cell labeling to illuminate the morphology of neurons and glia with cre-dependent morf mice. *Neuron*, 108(1):111–127.e6.

Wang, X.-J. (2020). Macroscopic gradients of synaptic excitation and inhibition in the neocortex. *Nature Reviews Neuroscience*, 21(3):169–178.

Winnubst, J., Bas, E., Ferreira, T. A., Wu, Z., Economo, M. N., Edson, P., Arthur, B. J., Bruns, C., Rokicki, K., Schauder, D., Olbris, D. J., Murphy, S. D., Ackerman, D. G., Arshadi, C., Baldwin, P., Blake, R., Elsayed, A., Hasan, M., Ramirez, D., Santos, B. D., Weldon, M., Zafar, A., Dudman, J. T., Gerfen, C. R., Hantman, A. W., Korff, W., Sternson, S. M., Spruston, N., Svoboda, K., and Chandrashekar, J. (2019). Reconstruction of 1,000 projection neurons reveals new cell types and organization of long-range connectivity in the mouse brain. *Cell*, 179(1):268 – 281.e13.

Woolsey, T. A. and Van der Loos, H. (1970). The structural organization of layer iv in the somatosensory region (s i) of mouse cerebral cortex: The description of a cortical field composed of discrete cytoarchitectonic units. *Brain Research*, 17(2):205–242.
